# New thioxothiazolidinyl-acetamides derivatives as potent urease inhibitors: design, synthesis, in vitro inhibition, and molecular dynamic simulation

**DOI:** 10.1038/s41598-022-27234-3

**Published:** 2023-01-02

**Authors:** Navid Dastyafteh, Milad Noori, Mohammad Nazari Montazer, Kamiar Zomorodian, Somayeh Yazdanpanah, Aida Iraji, Minoo Khalili Ghomi, Shahrzad Javanshir, Mehdi Asadi, Mehdi Dianatpour, Mahmood Biglar, Bagher Larijani, Massoud Amanlou, Mohammad Mahdavi

**Affiliations:** 1 Endocrinology and Metabolism Research Center, Endocrinology and Metabolism Clinical Sciences Institute, Tehran University of Medical Sciences, Tehran, Iran; 2 Department of Medicinal Chemistry, Faculty of Pharmacy, Tehran University of Medical Sciences, Tehran, Iran; 3 Department of Medical Mycology and Parasitology, School of Medicine, Shiraz University of Medical Sciences, Shiraz, Iran; 4 Stem Cells Technology Research Center, Shiraz University of Medical Sciences, Shiraz, Iran; 5 Central Research Laboratory, Shiraz University of Medical Sciences, Shiraz, Iran; 6 Department of Chemistry, Iran University of Science and Technology, Tehran, Iran; 7 Drug Design and Development Research Center, The Institute of Pharmaceutical Sciences (TIPS), Tehran University of Medical Sciences, Tehran, Iran

**Keywords:** Chemical biology, Drug discovery

## Abstract

To identify potent urease inhibitors, in the current study, a series of thioxothiazolidinyl-acetamides were designed and synthesized. The prepared compounds were characterized by spectroscopic techniques, including FTIR, ^1^HNMR, ^13^CNMR, and elemental analysis. In the enzymatic assessments, it was demonstrated that all derivatives had significant urease inhibition with IC_50_ values in the range of 1.473–9.274 µM in comparison with the positive control hydroxyurea (IC_50_ = 100.21 ± 2.5 µM) and thiourea (IC_50_ = 23.62 ± 0.84 µM). Compound 6i (N-benzyl-3-butyl-4-oxo-2-thioxothiazolidine-5-carboxamide) was the most active agent with an IC_50_ value of 1.473 µM. Additionally, kinetic investigation and in silico assessments of 6i was carried out to understand the type of inhibition and behavior of the most potent derivative within the binding site of the enzyme. Noteworthy, the anti-urease assay against *P. vulgaris* revealed 6e and 6i as the most active agents with IC_50_ values of 15.27 ± 2.40 and 17.78 ± 3.75 µg/mL, respectively. Antimicrobial evaluations of all compounds reveal that compounds 6n and 6o were the most potent antimicrobial agents against the standard and resistant *S. aureus*. 6n and 6o also showed 37 and 27% inhibition in the development of biofilm by *S. aureus* at 512 µg/ml. Furthermore, the MTT test showed no toxicity up to 100 µM. Taken together, the study suggests that the synthesized thioxothiazolidinyl-acetamides bases derivatives may serve as potential hits as urease inhibitors.

## Introduction

Urease (EC 3.5.1.5), a nickel-containing enzyme, belongs to the superfamily of amidohydrolases and phosphotriesterase which catalyzes the hydrolysis of urea into ammonia (NH_3_) and carbamate in living systems at approximately 10^14^ times faster than an uncatalyzed reaction^1^. The increase in pH through the increase in the amounts of NH_3_ causes health complications in humans and animals, including kidney stone formation, pyelonephritis, hepatic encephalopathy, and hepatic coma^2^.

*Klebsiella aerogenes*, *Bacillus pasteurii*, *Proteus vulgaris* as well as *Helicobacter pylori* (*H. pylori*) are just some examples of urease-positive microorganisms^3–5^. Noteworthy, one of the major public health problems is related to *H. pylori* presented in approximately 50% of the world's population^6^. *H. pylori* is a gram-negative urease-positive bacterial that survives in an acidic environment, such as the stomach (pH = 1–2)^2^. *H. pylori* infections induce gastric inflammation and increase the risk of developing gastric ulcers and gastric adenocarcinoma^6^. Urease as a virulence factor represents up to 10% of the total protein content of *H. pylori* which helps to colonize microorganisms. Also, increasing urease amounts is a great danger to environmental safety and imposes great economical burdens due to fast growth and the production of high amounts of toxic ammonia in biological systems^7,8^.

**Figure 1:**
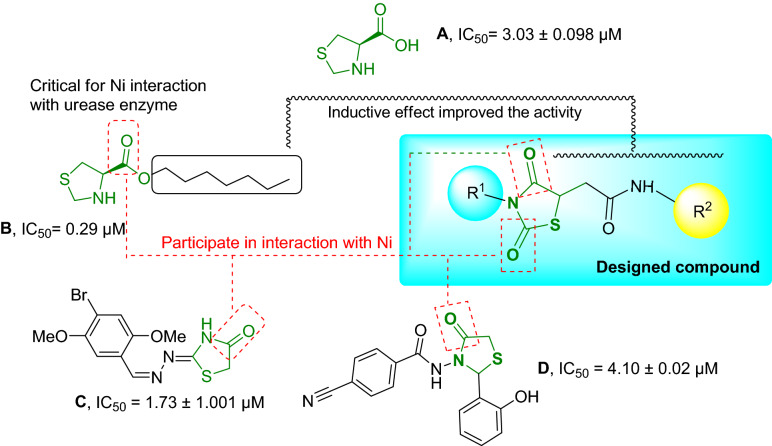
Rational design for the synthesis of novel thioxothiazolidinyl-acetamide as the urease inhibitor.

The binding site of urease contains two Ni atoms linked to hydroxide ions and three water molecules. Urea molecule as the substrate of enzyme participates in weak H-bound interactions with enzyme and breaks into ammonia and carbonic acid^9^. According to the structure of the urease binding site, strategies to design urease inhibitors have received considerable attention from the scientific community, which may be an effective therapy against diseases caused by urease-dependent pathogenic microorganisms in recent years. The diverse range of synthetic compounds has been designed to impede the growing challenges related to ureolytic microorganisms, including thioureas^13^ triazoles, thiadiazoles^10^, benzimidazoles^11^, hydroxamic acid^12^, phosphoramidate^13^, and thiazolacetamide^14^. Among the different derivatives understudy, thiazolidine derivatives showed remarkable enzyme inhibition potential. Virtual screening on the internal combinatorial library among 90,000 ligands introduced compound A (Fig. 1.) as a potent urease inhibitor. The molecular docking study showed that the configuration of the stereo-center at position 4 of the thiazolidine ring enormously affects the urease inhibition activity^15^. Lodhi et al.^16^ introduced a series of thiazolidine esters (Fig. 1. Compound B) as potential anti-urease agents. Results showed that compound B with heptyl ester was the most active inhibitor of the enzyme. In silico assessments exhibited the critical role of carbonyl in forming a pseudotetrahedral geometry responsible for the principal interaction with Ni of the urease. The authors deduced that the increase in the activity could be due to the influence of inductive effects than steric hindrance. In another study, a series of 4-thiazolidinone analogs with varying degrees of urease inhibitory potential (IC_50_ = 1.73–69.65 µM) were designed. A molecular docking study of the most potent derivative (Compound **C**) showed that the carbonyl of the thiazolidine ring coordinated with both nickel ions^17^. In 2019, Schiff base-thiazolidinones were developed by Taha et al. group. The most potent derivative (Fig. 1. Compound D) adopted hydrogen and hydrophobic interactions with the catalytic and the modified residues, such as H323, R339, M367, KCX-220, as well as ionic bonds with two embedded Ni ions of the active site.

Since strong binding to the nickel center is important for urease inhibition, the higher chelation properties might affect the urease inhibition. Conserving the important role of thioxothiazolidinyl acetamide to participate in different interactions, especially ionic and hydrogen bonds, such moieties might be helpful to improve urease inhibition, the primary molecular docking study mostly confirmed these assumptions. The designed compound showed a higher glide score (−11.185 kcal/mol) than compounds A, B, C, and D with glide score values in the range of −9.998 to −10.882 kcal/mol. Moreover designed compound demonstrated several hydrophobic interactions with the active site pocket of the enzyme alongside the H-bond interaction through its amide group and chelating properties, which altogether verified the high potential anti-urease activity of the designed compounds. As a result, in continuation to our previous efforts in developing urease inhibitors^18–20^, in the present study, we focused on designing and developing a series of thioxothiazolidinyl-acetamides derivatives.

## Results and discussion

### Chemistry

Synthesis of the target compounds, thioxothiazolidinyl -acetamide (6a-o) was schematically described in Fig. 2. Briefly, different amine (1) was added to CS_2_ (2) in H_2_O at room temperature for 20 min, followed by the addition of maleic anhydride (3). The reaction was stirred at room temperature for 5 h to synthesize compound 4. The corresponding derivatives (6a-o) were prepared by reacting derivative 4 with different amine derivatives (5) in anhydrous DMF in the presence of DIPEA and TBTU at room temperature for 5 h. The reaction was diluted with water, and the resulting precipitate was collected by filtration. All synthesized compounds were characterized by FTIR, ^1^H-NMR, ^13^C-NMR, and elemental analysis. ^1^H NMR and ^13^C NMR spectra for compounds 6a–o are available in Supplementary Materials (Figures S1–30).

**Figure 2:**
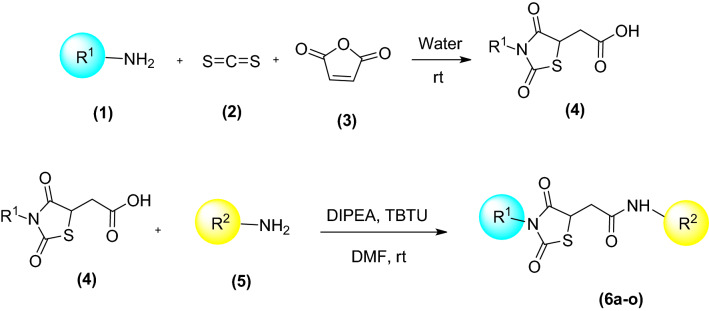
Synthesis of thioxothiazolidinyl-acetamide compounds (6a–o).

### In vitro urease inhibition

Fifteen thioxothiazolidinyl-acetamides derivatives (6a–o) were synthesized as urease inhibitors. These derivatives exhibited significant urease inhibition with IC_50_ values ranging between 1.47 and 9.274 μM when compared with the standard inhibitor hydroxyurea with an IC_50_ value of 100 μM (Table 1).

To explain the structure–activity relationships (SARs), synthesized hybrids were divided into three categories based on the presence of different moieties at the R^1^ position.

Among the first set of derivatives (6a–h) bearing benzyl at the R^1^ position, all compounds demonstrated significant inhibitory potency with IC_50_ values in the range of 1.612–4.019 μM. In detail, among the aromatic substitutions at R^2^ position, the following order to potency was seen so that 6c (R = *para-*fluorobenzyl) ≥ 6a (R = benzyl) ˃ 6b (R = *para*-methylbenzyl). Replacement of aromatic substitution with aliphatic group resulted in 6d–h in which overall improvement in the potency was observed (IC_50_ values in the range of 1.596–1.862 μM). Although there are no significant differences among 6d–h, compound 6f. with isobutyl substituent on the benzyl ring showed the most potent inhibitory activity among benzyl-containing compounds at R^1^ followed by 6 g.

Assessments on 6i-k derivatives (R^1^ = butyl) showed that 6i as an unsubstituted derivative (R^1^ = benzyl) in this group showed an IC_50_ value of 1.473 µM. The presence of methyl as an electron-donating group on the benzyl ring (6j) deteriorated the inhibitory potency. Although the presence of isobutyl at R^2^ improved the activity compared to 6j, it was inferior activity compared to 6i.

By comparing the IC_50_ values in 6 l–o, it can be implied that the least potency belongs to this group, so 6 l as an unsubstituted derivative recorded an IC_50_ value of 4.397 μM. Unlike the previous sets, the *para*-methylbenzyl group (electron-donating group) improved the activity compared to 6 l. The least potent inhibitor among all synthesized compounds was 6o (R^2^: propenyl) still demonstrated tenfold improvement in the activity compared to hydroxyurea as a positive control.

Afterward, the effect of the same moieties at R^2^ was explored. The tested compounds bearing benzyl at R^2^ showed that the linear aliphatic chain (6i) was more favorable compared to aromatic (6a) and branch aliphatic (6 l) groups. However, different trends were seen in derivatives bearing the *para*-methylbenzyl at R^2^ so that branch aliphatic (6 m) showed better inhibitory activity followed by aromatic (6b) and linear aliphatic (6i) moieties.

In line with the current study, assessments of arylhydrazide bearing thiazolidinone showed significant IC_50_ values ranging between 4.10 ± 0.02 and 38.20 ± 1.10 μM. SARs showed the presence of two chloro groups at *ortho* and *para* positions improved the potency^21^. 4-Thiazolidinone analogs evaluations against urease exhibited varying degrees of urease inhibitory potential with IC_50_ values 1.73–69.65 µM. SARs showed that both electrons donating as well as electron-withdrawing groups on the phenyl ring play role in the inhibition but the electron-donating groups are superior up to some extent^17^. Also in the other study, it was shown that all branched analogs of thiazolidine ester are less active than their straight-chain analogs, it may be probably because of the steric bulk of branched-chain substituents^16^.

### Enzyme kinetic studies

**Figure 3:**
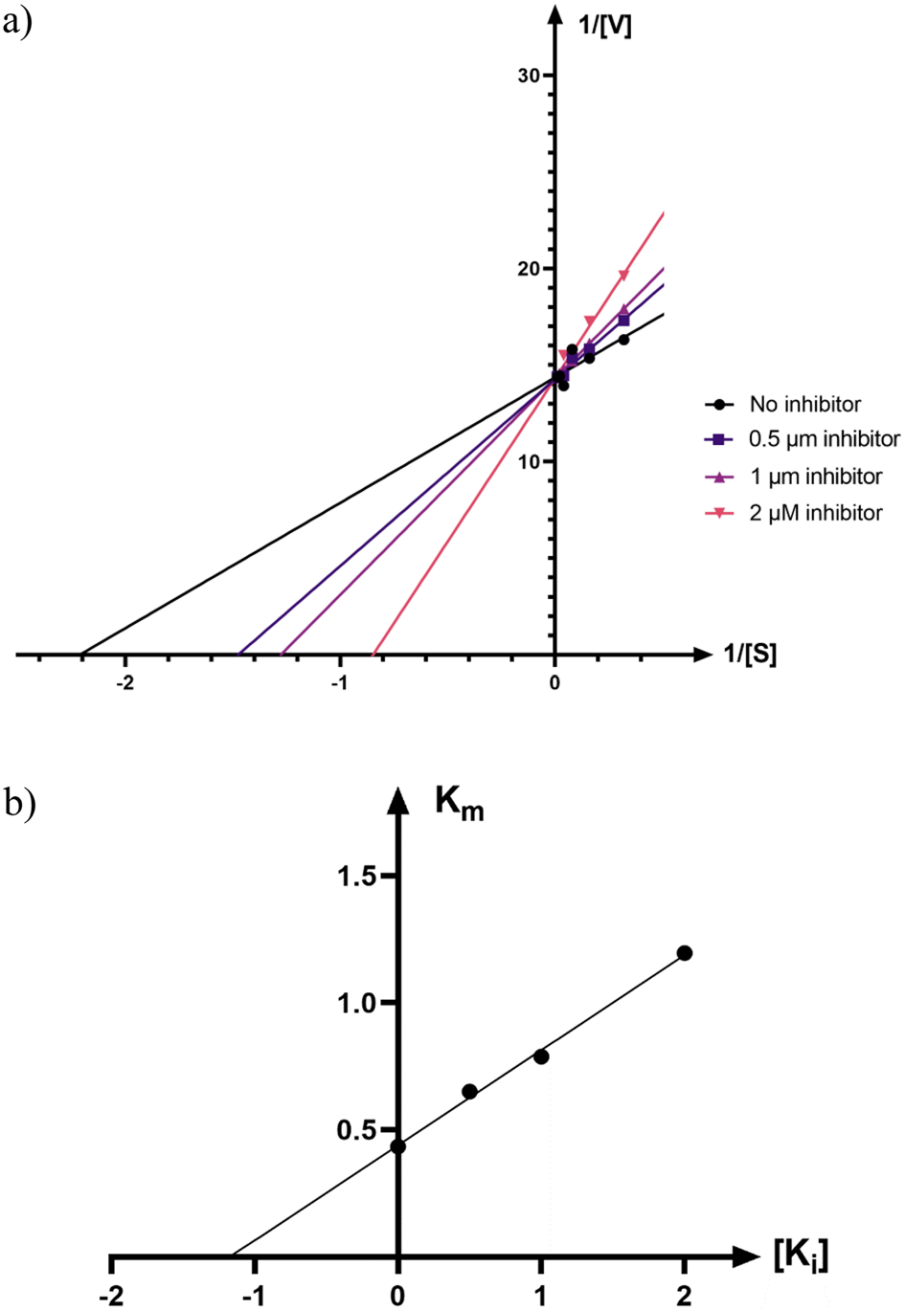
Kinetics of urease inhibition by 6i. (**a**) The Lineweaver–Burk plot in the absence and presence of different concentrations of 6i; (**b**) The secondary plot between *K*_m_ and various concentrations of 6i.

According to Fig. 3. the Lineweaver–Burk plot showed that the *K*_m_ gradually increased and *V*_*max*_ remained unchanged with increasing 6i concentration, indicating a competitive inhibition. The results showed that 6i binds to the active site on the enzyme and competed with the substrate to bind to the active site (Fig. 3a). Furthermore, the plot of the *K*_m_ versus different inhibitor concentrations gave an estimate of the inhibition constant, *K*_i_ of 1.173 µM (Fig. 3b).

### Molecular modeling study

**Figure 4:**
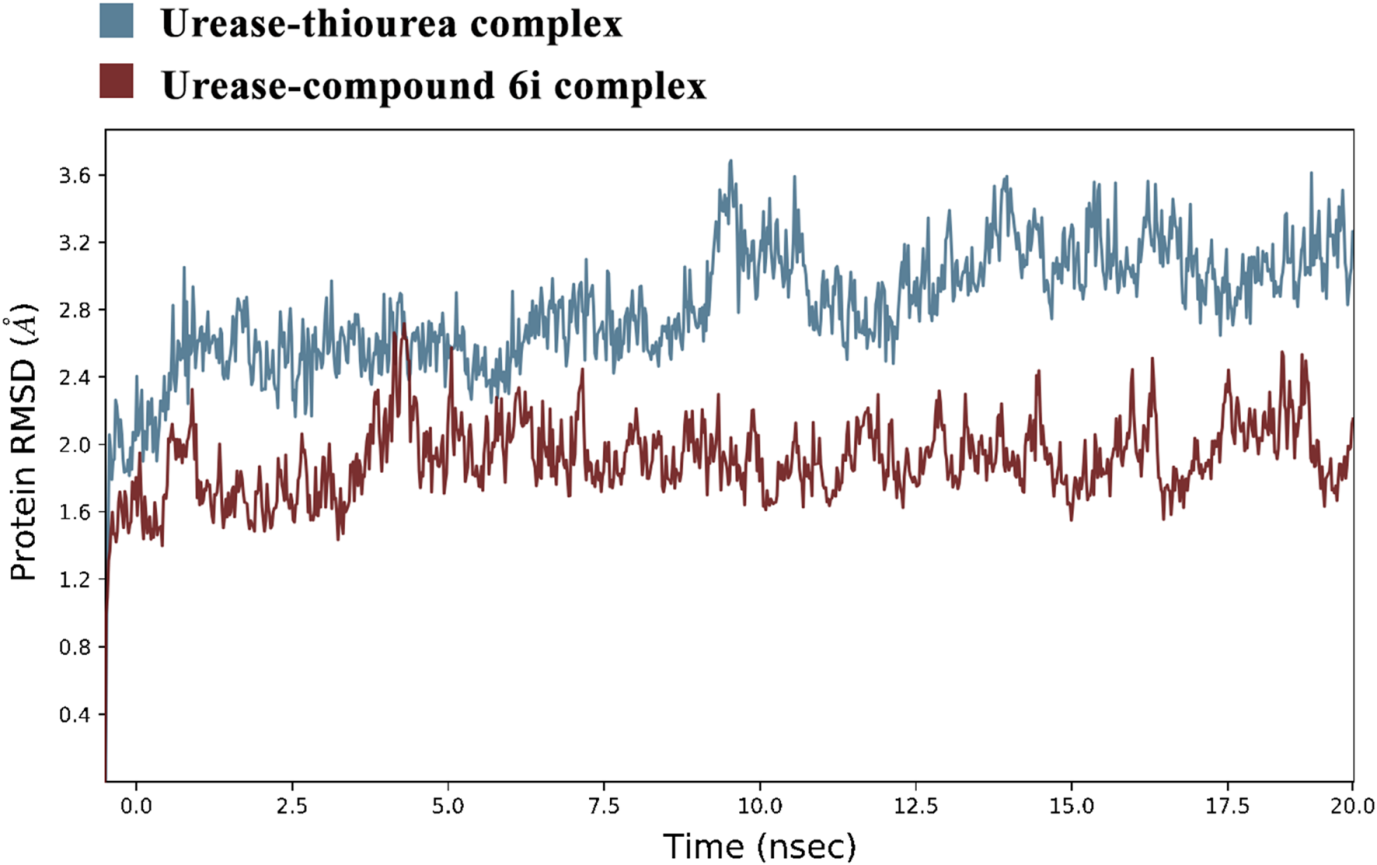
RMSD plots of the urease backbone complexed with compound 6i (red) and thiourea (blue).

Urease consists of four main domains, and the active site of urease is located in (αβ)8 TIM barrel domain. In the active site, two nickel ions are bridged by H-bonds of the carbamylated lysine (KCX 490). Other coordinating residues such as His407, His409, Asp633, KCX490, His519, His545, and Gly550 are vital for the urease enzyme activity. Furthermore, the residues of the flap pocket (590–608) play a critical role in the hydrolysis process. To study the steadiness of the protein–ligand complex, the root mean square deviation (RMSD) of the complexed backbone was investigated in MD simulation. The RMSD plot of the urease enzyme backbone in complex with the compound 6i and in complex with the thiourea was demonstrated in Fig. 4. Changes of the order of 1–3 Å are perfectly acceptable for small, globular proteins. Changes much larger indicate the protein is undergoing a large conformational change during the simulation. Based on the ligand-complex RMSD result (Fig. 4), the engaged simulation period was adequate to reach a balanced structure over the simulation time. Also, compound 6i reached stability after 5 ns while thiourea complex reached overall stability after 10 ns. Furthermore, the fluctuation of compound 6i (average of 2 Å) seemed to be significantly lower than the thiourea complex (average of 3.1 Å).

**Figure 5:**
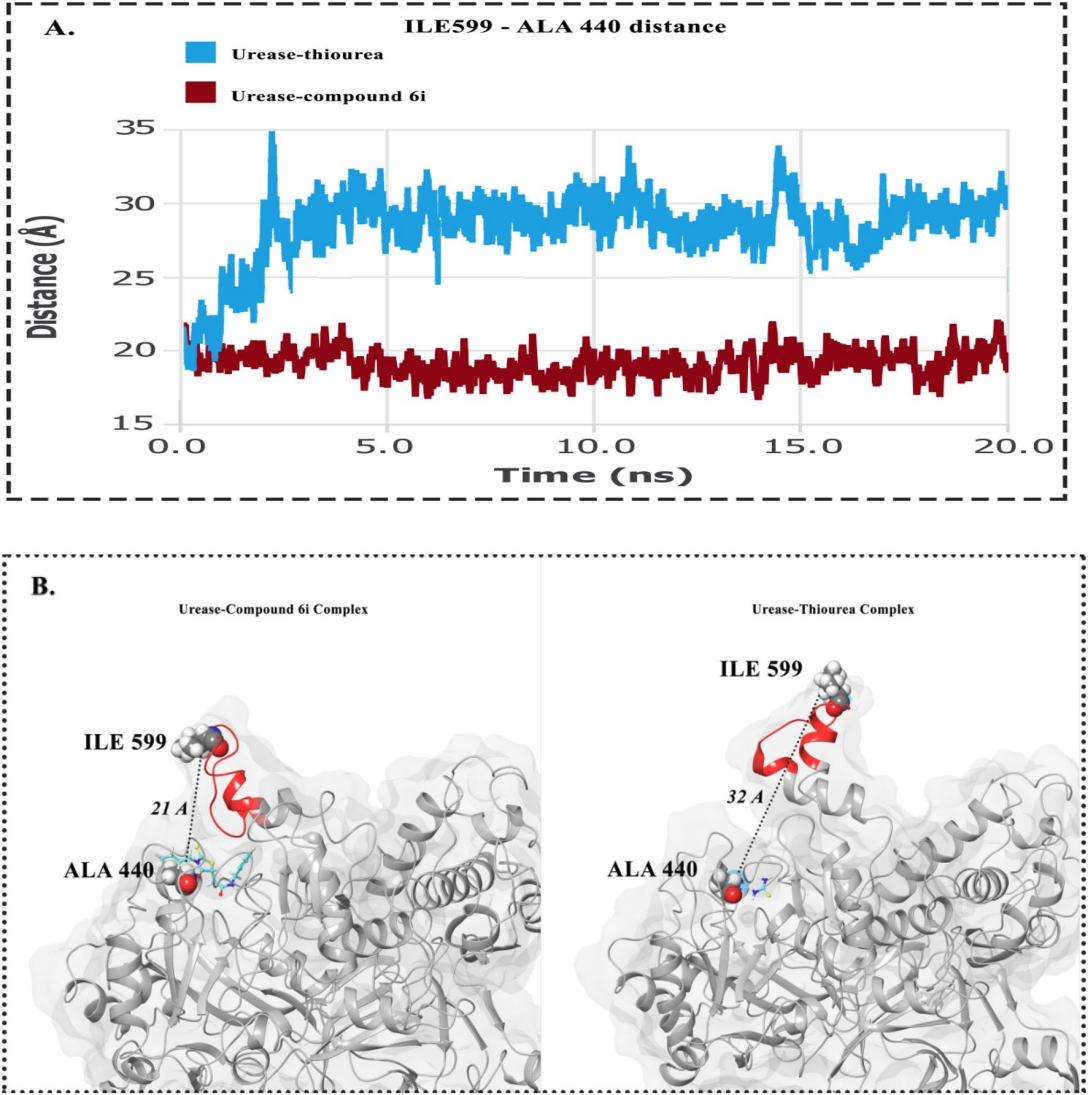
(**A**) Distance of Ile599-Ala440 compared to simulation time in urease-thiourea complex (blue) and urea-compound 6i complex (red) (**B**) Schematic view of flap (red) residues in open and closed states.

Studies suggest the active site of jack bean urease includes a dynamic flap consisting of a helix-turn-helix structure in the region of Met590 to His607 amino acids^22^. The flap mechanism in the activity of the enzyme is to rather cover or uncover the enzyme's active site pocket which in the closed state of the flap the physical accessibility of the substrate to the enzyme's active site pocket would be restricted. As is shown in Fig. 5, the distance between Ile599-Ala440 as a criterion of flap distance to the active site indicates that compound 6i successfully closed the active site flap with an average distance of 21 Å compared to the open flap in the complex of enzyme-thiourea with the average distance of 32 Å.

**Figure 6:**
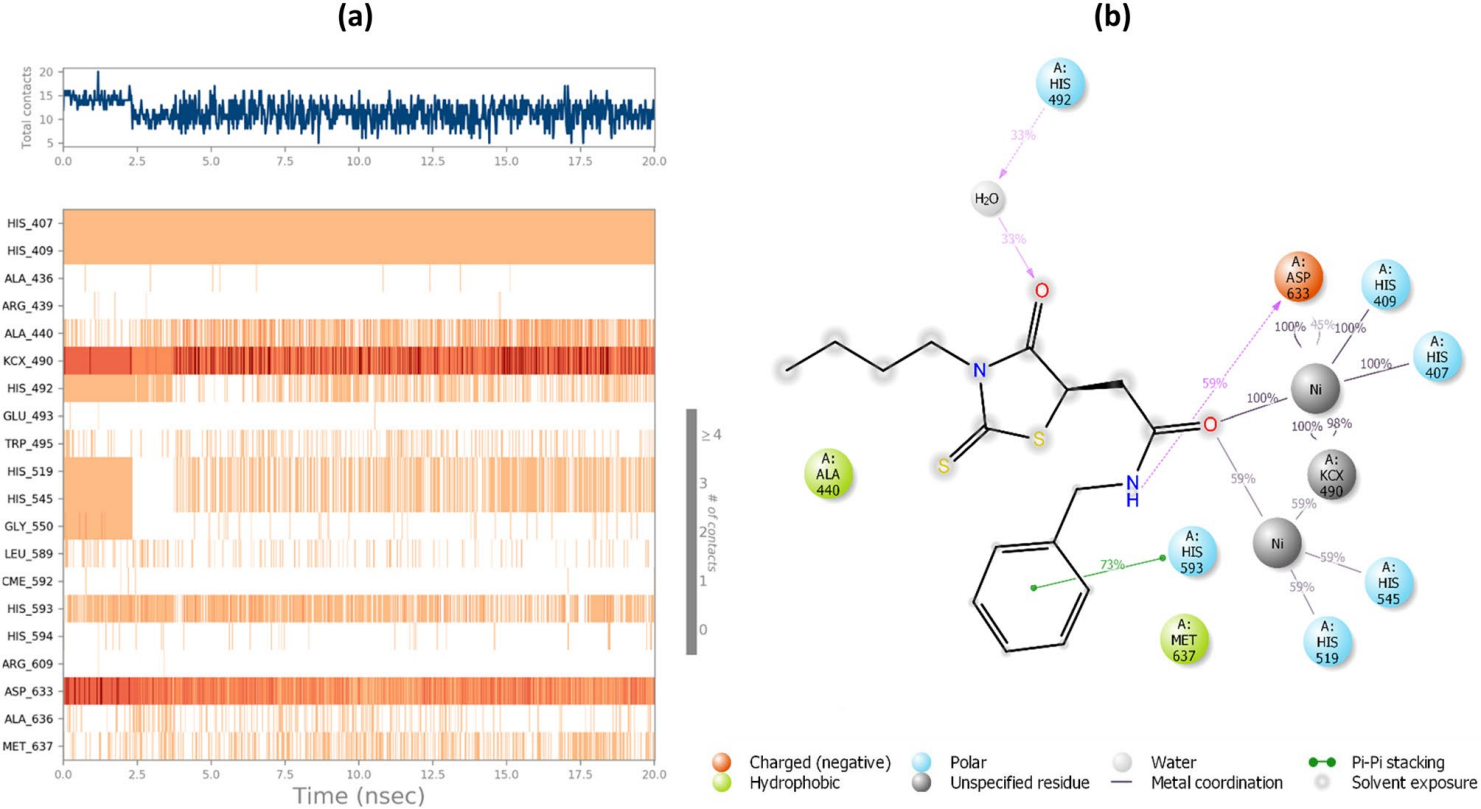
Timeline rendering of interacting residues during the whole simulation time in urease complexed with compound 6i (**a**). 2D representation of ligand-residue interactions (**b**).

Interactions that occur more than 30.0% of the simulation time in the selected trajectory 0.00 through 20.02 ns) are shown in Fig. 6. As can be seen, derivative 6i formed metal coordinate interactions with Ni and stabilized through interactions with His407, His409, KCX490, His519, and His545. The thioxothiazolidine ring provided interactions with His492 via a water bridge. Amide linker participated in H-bound interaction with Asp633, and benzyl moiety formed pi-pi stacked interaction with His593.

### Urease inhibitory activity of tested compounds against *P. vulgaris*

The urease inhibitory activity of some derivatives against *P. vulgaris* was measured spectroscopically at 560 nm. Interesting results were obtained so that 6i (R^1^: benzyl, R^2^: butyl) and 6e (R^1^: butyl, R^2^: benzyl) bearing the same substitution at various positions demonstrated approximately the same urease inhibition. Assessments on the tested derivatives bearing benzyl pendant at R^1^ exhibited the following order of potency at R^2^ so that n-butyl (6e) > isobutyl (6f.) > propenyl (6 g) > propyl (6d) > cyclopentyl (6i). It seems that four-carbon alkyl substituents significantly improved the activity, followed by the three-carbon alkyl group. However, the presence of the aliphatic ring at the R^2^ position deteriorated the activity, so the 6 h derivative demonstrated 48.45% inhibition at 512 µg/ml (Table 2).

**Table 1:**
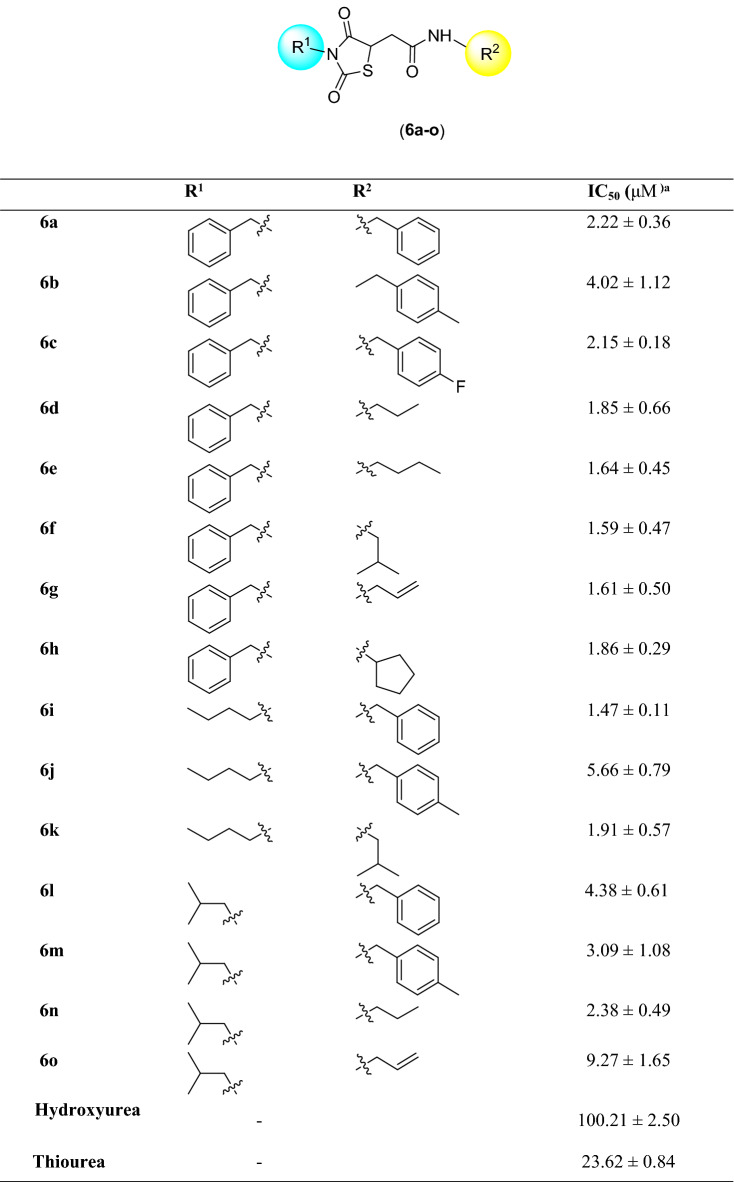
Urease inhibitory activity of compounds 6a-n.

^a^Values are the mean ± SEM. All experiments were performed at least three times.

### Antimicrobial activity study

The antimicrobial activity of all synthetic compounds is shown in Table 3. Among the tested compounds, 6n (R^1^: isobutyl, R^2^: propenyl) and 6o (R^1^: isobutyl, R^2^: propyl) inhibited the growth of *S. aureus* at a concentration of 128 µg/mL. Moreover, the minimum inhibitory concentration (MIC) value of compound 6o against *E.coli* was 512 µg/mL. Both mentioned derivatives contain isobutyl moiety at R^1^ as well as a linear chain at R^2^. Other compounds did not exhibit antimicrobial activities at a concentration up to 512 µg/mL.

**Table 2:** Urease inhibitory activity of selected compounds against *P. vulgaris.*

Compound	IC_50_ (µg/ml) of *P. vulgaris*
6d	125.89 ± 8.65
6e	15.27 ± 2.40
6f.	32.36 ± 5.26
6 g	61.66 ± 6.41
6 h	≥ 512
6i	17.78 ± 3.75

According to the obtained results, overall, derivatives had no antimicrobial effects on the tested microorganisms. The exception in this trend came back to 6n and 6o. It seems that the high activity of tested compounds against ureolytic microorganisms strengthens the hypothesis that this scaffold is an ideal pharmacophore as a urease inhibitory agent.

Regarding the antibacterial activity of 6n and 6o against the standard strain of *S. aureus*, these derivatives were tested against methicillin-resistant *Staphylococcus aureus* (MRSA) isolates. Antimicrobial activities against resistant bacterium exhibited MIC values of 128 and 512 µg/ml for 6n and 6o, respectively.

### Inhibition of biofilm formation

The biofilm formation in the presence of 6n and 6o derivatives as the most potent compounds were evaluated. According to the results, 37 and 27% inhibition in the development of biofilm by *S. aureus* were observed at 512 µg/ml of tested compounds. These results indicate that 6n and 6o derivatives had limited antibiofilm activity against *S. aureus.* The absence of antibiofilm activity for antimicrobial agents is evidence that cells in a biofilm structure are more resistant to antimicrobial agents compared to planktonic cells^23^.

### Cytotoxic activity

Finally, the toxicity of 6a–o derivatives against MOLT-4 cells was determined by the MTT assay. As can be seen in Table 4, no cytotoxicity was recorded up to 100 μM concentration.

**Table 3:** Antimicrobial activity of thioxothiazolidinyl-acetamide compounds (6a–o).

	Microorganism			
	*S. aureus* ATCC 25923	*E. coli* ATCC 25922	*P. vulgaris*	*C. albicans* ATCC 10261
Compounds	MIC* MBC*	MIC* MBC*	MIC* MBC*	MIC* MFC*
6a	> 512 ND	> 512 ND	> 512 ND	> 512 ND
6b	> 512 ND	> 512 ND	> 512 ND	> 512 ND
6c	> 512 ND	> 512 ND	> 512 ND	> 512 ND
6d	> 512 ND	> 512 ND	> 512 ND	> 512 ND
6e	> 512 ND	> 512 ND	> 512 ND	> 512 ND
6f.	> 512 ND	> 512 ND	> 512 ND	> 512 ND
6 g	> 512 ND	> 512 ND	> 512 ND	> 512 ND
6 h	> 512 ND	> 512 ND	> 512 ND	> 512 ND
6i	> 512 ND	> 512 ND	> 512 ND	> 512 ND
6 g	> 512 ND	> 512 ND	> 512 ND	> 512 ND
6 k	> 512 ND	> 512 ND	> 512 ND	> 512 ND
6 l	> 512 ND	> 512 ND	> 512 ND	> 512 ND
6 m	> 512 ND	> 512 ND	> 512 ND	> 512 ND
6n	128 > 512	> 512 ND	> 512 ND	> 512 ND
6o	128 256	512 > 512	> 512 ND	> 512 ND
Ciprofloxacin/Fluconazole	0.5 ND	0.25 ND	1 ND	4 ND

*MIC Minimum Inhibitory Concentration, *MBC/MFC* Minimum Bactericidal/Fungicidal, MICs and MBCs are expressed as µg/ml.

**Table 4:** The viability percent of MOLT-4 cells after being exposed to thioxothiazolidinyl-acetamide compounds (6a–o).

Compound	% Viability at 100 µM	Compound	% Viability at 100 µM
6a	95.5 ± 2.7	6i	97.8 ± 2.5
6b	91.2 ± 4.2	6j	97.7 ± 4.6
6c	92.7 ± 3.6	6 k	98.5 ± 1.4
6d	95.5 ± 3.2	6 l	93.6 ± 4.5
6e	91.7 ± 2.8	6 m	97.7 ± 2.9
6f.	91.9 ± 1.3	6n	96.4 ± 2.2
6 g	91.6 ± 3.7	6o	97.3 ± 3.5
6 h	95.0 ± 4.2	Doxorubicin *	12.7 ± 2.4

*The results of doxorubicin were reported in the term of IC_50_ (µM).

## Conclusion

In conclusion, a novel series of thioxothiazolidinyl-acetamides 6a–o were rationally designed and synthesized. All derivatives evaluated against urease and IC_50_ values in the range of 1.473–9.274 µM were recorded compared to the positive control hydroxyurea (IC_50_ = 100.21 ± 2.5 µM) and thiourea (IC_50_ = 23.62 ± 0.84 µM). SARs study established that the nature and the position of substitutions at R^1^ and R^2^ had crucial roles in defining the urease inhibition activity. In addition, compound 6i as the most potent derivative demonstrated the competitive type of inhibition in the enzymatic assay. Furthermore, in silico evaluations on 6i showed polar and nonpolar contacts with the crucial residues in the binding site of urease and Ni cofactors.

The anti-ureolytic assay showed significant potency of these derivatives against *P.vulgaris* in the microbial assay. Among them, compounds 6e and 6i exhibited the most potent inhibitory effect against urease with an IC_50_ value of 15.27 ± 2.40 and 17.78 ± 3.75 µg/mL, respectively. Consequently, antimicrobial assessments of these derivatives against the standard and resistant species showed moderate antimicrobial activity of 6n and 6o derivatives. In addition, these analogs were analyzed for their cytotoxicity and revealed no toxicity against MOLT-4 cell lines. Regarding the high potencies of these derivatives as anti-urease agents, the design and synthesis of new thioxothiazolidinyl bearing candidates will require shedding more light on the underlying SARs that account for the biological activity.

## Experimental

### Chemistry

#### N-benzyl-2-(3-benzyl-4-oxo-2-thioxothiazolidin-5-yl)acetamide (6a)

Brown solid; Yield: 70%; MP = 168–170 °C; IR (KBr, v_max_) 3350 (NH), 3032 (C-H Aromatic), 2984 (CH_2_ Aliphatic), 1690 (C=O) Cm^−1^; ^1^H NMR (400 MHz, DMSO-d_6_) δ 8.65 (t, ^3^*J*_*H,H*_ = 5.9 Hz, 1H, NH), 7.37–7.23 (m, 10H, 2 × Ph), 5.16 (d, ^3^*J*_*H,H*_ = 15.1 Hz, 1H, H_A__Diastereotopic_ CH_2_-Ph), 5.04 (d, ^3^*J*_*H,H*_ = 15.1 Hz, 1H, H_B__Diastereotopic_, CH_2_-Ph) 4.87 (dd, ^3^*J*_*H,H*_ = *8.7,* ^2^*J*_*H,H*_ = *3.9 Hz*, 1H, CH_Chiral_-CH_2_), 4.29 (d, ^3^*J*_*H,H*_ = 5.9 Hz, 2H, CH_2_-NH), 3.16 (dd, ^*3*^*J*_*H,H*_ = 16.7,8.7, ^2^*J*_*H,H*_ = 4 Hz,1H, H_A__Diastereotopic_, CH_2_-CH_Chial_), 3.02 (dd, ^3^J_H,H_ = 16.6 Hz, ^2^*J*_*H,H*_ = 4 Hz, 1H, H_B__Diastereotopic_, CH_2_-CH_Chial_) ppm. ^13^C NMR (101 MHz, DMSO-d_6_): δ 203.73, 176.96, 168.92, 164.18, 139.46, 139.41, 135.56, 133.28, 128.85, 128.80, 127.94, 127.88, 127.76, 127.33, 47.50, 47.10, 42.72, 36.87, ppm; ESI–MS (C_19_H_18_N_2_O_2_S_2_): Calcd: m/z 370.08 [M + H]^+^, observed m/z 370.15 [M + H]^+^; Anal. Calcd: C_19_H_18_N_2_O_2_S_2_: C, 61.60; H, 4.90; N, 7.56; Found C, 61.80; H, 5.10; N, 7.75.

#### 2-(3-benzyl-4-oxo-2-thioxothiazolidin-5-yl)-N-(4-methylbenzyl)acetamide (6b)

Brown solid; Yield: 83%; MP = 167–169 °C; IR (KBr, v_max_) 3355 (NH), 3036 (C–H Aromatic), 2978(CH_2_ Aliphatic), 1685 (C=O) Cm^−1^; ^1^H NMR (400 MHz, DMSO-d_6_) δ 8.60 (t, ^3^*J*_*H,H*_ = 6.0 Hz, 1H, NH), 7.37–7.10 (m, 10H, 2 × Ph), 5.16 (d, ^3^*J*_*H,H*_ = 15.1 Hz,1H, H_A__Diastereotopic_ CH_2_-Ph), 5.04 (d, ^3^*J*_*H,H*_ = 15.1 Hz, 1H, H_B__Diastereotopic,_ CH_2_-Ph) 4.86 (dd, ^3^*J*_*H,H*_ = *8.7,* ^2^*J*_*H,H*_ = *3.9 Hz*, 1H, CH_Chiral_-CH_2_), 4.24 (d, ^3^*J*_*H,H*_ = 5.8 Hz, 2H, CH_2_-NH), 3.15 (dd, ^*3*^*J*_*H,H*_ = 16.6 Hz, ^2^*J*_*H,H*_ = 4 Hz, 1H, H_A__Diastereotopic_, CH_2_-CH_Chial_), 3.00 (dd, ^*3*^*J*_*H,H*_ = 16.6 Hz, ^2^*J*_*H,H*_ = 4 Hz,1H, H_B Diastereotopic_, CH_2_-CH_Chial_), 2.28 (s, 3H, CH_3_) ppm. ^13^C NMR (101 MHz, DMSO-*d*_*6*_): δ 203.78, 176.96, 168.82, 139.47, 136.42, 135.57, 129.33, 128.85, 128.80, 127.94, 127.88, 127.77, 127.37, 47.50, 47.10, 42.49, 36.88, 21.14 ppm; Anal. Calcd: C_20_H_20_N_2_O_2_S_2_: C, 62.47; H, 5.24; N, 7.29; Found C, 62.65; H, 5.44; N, 7.50.

#### 2-(3-benzyl-4-oxo-2-thioxothiazolidin-5-yl)-N-(4-fluorobenzyl)acetamide (6c)

Brown solid; Yield: 76%; MP = 169–171 °C; IR (KBr, v_max_) 3340 (NH), 3025 (C–H Aromatic), 2985 (CH_2_ Aliphatic), 1665 (C=O) Cm^−1^; ^1^H NMR (400 MHz, DMSO-*d*_*6*_) δ 8.60 (t, ^3^*J*_*H,H*_ = 5.96 Hz, 1H, NH), 7.38–7.10 (m, 10H, 2 × Ph), 5.16 (d, ^3^*J*_*H,H*_ = 15.0 Hz, 1H, H_A__Diastereotopic_ CH_2_-Ph), 5.04 (d, ^3^*J*_*H,H*_ = 15.0 Hz, 1H, H_B__Diastereotopic,_ CH_2_-Ph), 4.86 (dd, ^3^*J*_*H,H*_ = *8.6,* ^2^*J*_*H,H*_ = *3.8 Hz*, 1H, CH _Chiral_-CH_2_), 4.27 (d, ^3^*J*_*H,H*_ = 5.9 Hz, 2H, CH_2_-NH), 3.15 (dd, ^*3*^*J*_*H,H*_ = 16.6 Hz, ^2^*J*_*H,H*_ = 8.7 Hz, 1H, H_A__Diastereotopic_, CH_2_–CH _Chiral_), 3.0 (dd, ^*3*^*J*_*H,H*_ = 16.6 Hz, ^2^*J*_*H,H*_* 8.7* Hz,1H, H_B__Diastereotopic_, CH_2_-CH_Chiral_) ppm. ^13^C NMR (101 MHz, DMSO-*d*_*6*_): δ 203.73, 176.96, 168.92, 162.89, 160.48, 135.71, 135.68, 135.56, 129.79, 129.71, 128.85, 128.79, 127.94, 127.88, 127.77, 127.37, 115.62, 115.40, 47.50, 47.07, 42.73, 42.01, 36.85 ppm; Anal. Calcd: C_19_H_17_FN_2_O_2_S_2_: C, 58.74; H, 4.41; N, 7.21; Found C, 58.95; H, 4.60; N, 7.31.

#### 2-(3-benzyl-4-oxo-2-thioxothiazolidin-5-yl)-N-propylacetamide (6d)

Brown solid; Yield: 93%; MP = 165–167 °C; IR (KBr, v_max_) 3335 (NH), 3050 (C-H Aromatic), 2945 (CH_2_ Aliphatic), 1675 (C=O) Cm^−1^; ^1^H NMR (400 MHz, DMSO-*d*_*6*_) δ 8.11 (t, ^3^*J*_*H,H*_ = 5.6 Hz, 1H, NH), 7.36–7.22 (m, 5H, Ph), 5.15 (d, ^3^*J*_*H,H*_ = 15.1 Hz,1H, H_A__Diastereotopic_ CH_2_-Ph), 5.03 (d, ^3^*J*_*H,H*_ = 15.1 Hz, 1H, H_B Diastereotopic,_ CH_2_-Ph), 4.82 (dd, ^3^*J*_*H,H*_ = *8.8,* ^2^*J*_*H,H*_ = *3.8* Hz, 1H, CH _Chiral_-CH_2_), 3.10–3.01 (m, 3H, CH_2__(1)__propyl_, CH_2_-CH_Chiral_), 3.01–2.88 (m, 1H, CH_2_–CH_Chial_), 1.40 (q, ^3^*J*_*H,H*_ = 7.2 Hz, 2H, CH_2__(2)__Propyl_), 0.84 (t, ^3^*J*_*H,H*_ = 7.4 Hz, 3H, CH_3 Propyl_) ppm. ^13^C NMR (101 MHz, DMSO-*d*_*6*_): δ 203.80, 176.97, 168.64, 135.57, 128.85, 127.93, 127.88, 127.86, 47.47, 47.11, 42.72, 40.93, 36.87, 22.75, 11.85 ppm; ESI–MS (C_15_H_18_N_2_O_2_S_2_): calculated m/z 322.08 [M + H]^+^, observed m/z 322.17 [M + H]^+^; Anal. Calcd: C_15_H_18_N_2_O_2_S_2_: C, 55.87; H, 5.63; N, 8.69; Found C, 56.05; H, 5.84; N, 8.86.

#### 2-(3-benzyl-4-oxo-2-thioxothiazolidin-5-yl)-N-butylacetamide (6e)

Cream solid;Yield:92%;MP = 166–168 °C IR (KBr, v_max_) 3345 (NH), 3030 (C-H Aromatic), 2975 (CH_2_ Aliphatic), 1650 (C=O) Cm^-1^; ^1^H NMR (400 MHz,DMSO-*d*_*6*_) δ 8.10 (t, ^*3*^*J*_*H,H*_ = 6.70, 1H, NH-CH_2_), 7.35–7.24 (m, 5H, Ph), 5.15 (d, ^*3*^*J*_*H,H*_ = 15.10 Hz, 1H, H_A__Diastropic_, CH_2_-Ph), 5.03 (d, ^*3*^*J*_*H,H*_ = 15.10 Hz, 1H, H_B Diastropic_, CH_2_-Ph), 4.81 (dd, ^*3*^*J*_*H,H*_ = *8.80, *^*2*^*J*_*H,H*_ = 3.80 Hz, 1H, CH _Chiral_-CH_2_), 3.10–3.01 (m, 3H, CH_2_-NH, H_A Diastropic_, CH_2_–CH_Chiral_), 2.95–2.86 (m, 1H, H_B Diastropic_, CH_2_-CH _Chiral_), 1.37 (q, ^*3*^*J*_*H,H*_ = 7.30 Hz, 2H, CH_2__butyl_-CH_2_), 1.28 (q, ^*3*^*J*_*H,H*_ = 7.30 Hz, 2H, CH_2__butyl_-CH_3_), 0.87 (t, ^*3*^*J*_*H,H*_ = 7.20 Hz, 3H, CH_3__butyl_-CH_2_). ppm. ^13^C NMR (101 MHz, DMSO-d6): δ 203.78, 176.96, 168.91, 168.59, 139.46, 135.58, 128.84, 128.79, 127.94, 127.87, 127.76, 127.37, 47.47, 47.12, 42.73, 38.81, 36.89, 31.56, 20.00, 14.11 ppm; ESI–MS (C_16_H_20_N_2_O_2_S_2_): calculated m/z 336.10 [M + H]^+^, observed m/z 336.18 [M + H]^+^; Anal. Calcd: C_16_H_20_N_2_O_2_S_2_; C, 57.11; H, 5.99; N, 8.33; Found C, 57.30; H, 6.10; N, 8.55.

#### 2-(3-benzyl-4-oxo-2-thioxothiazolidin-5-yl)-N-isobutylacetamide (6f)

Yellow solid; Yield: 78%; MP = 164–166 °C; IR (KBr, v_max_) 3330 (NH), 3046 (C-H Aromatic), 2950 (CH_2_ Aliphatic), 1670 (C=O) Cm^-1^; ^1^H NMR (400 MHz, DMSO-*d*_*6*_) δ 8.12 (t,* J* = 5.90 Hz, 1H, NH-CH_2 Isobutyl_), 7.37–7.22 (m, 5H, Ph), 5.15 (d, ^*3*^*J*_*H,H*_ = 15.10 Hz, 1H, H_A__diastropic_, CH_2_-Ph), 5.03 (d, ^*3*^*J*_*H,H*_ = 15.10 Hz, 1H, H_B__diastropic_, CH_2_-Ph), 4.82 (dd, ^*3*^*J*_*H,H*_ = 3.90, Hz, 1H, CH_chairal_-CH_2_), 3.15–3.03 (m, 1H, CH_2__Isobutyl_-NH), 3.00–2.85 (m, 3H, CH_2__Isobutyl_-NH, CH_2_–CH_Chiral_), 1.66 (d,t, ^*3*^*J*_*H,H*_ = 14.00, ^*2*^*J*_*H,H*_ = 7.10 Hz, 1H, CH _Isobutyl_), 0.89-0.087 (d, ^*3*^*J*_*H,H*_ = 6.2 Hz, 6H, 2 × CH_3__IsoPropyl_) ppm. ^13^C NMR (101 MHz, DMSO-*d*_*6*_): δ 203.76, 176.97, 168.92, 168.75, 139.46, 135.57, 128.85, 127.93, 127.76, 127.37, 47.46, 47.15, 46.65, 42.72, 36.91, 28.52, 20.54 ppm; ESI–MS (C_16_H_20_N_2_O_2_S_2_): calculated m/z 336.10 [M + H]^+^, observed m/z 336.16 [M + H]^+^; Anal. Calcd: C_16_H_20_N_2_O_2_S_2_: C, 57.11; H, 5.99; N, 8.33; Found C, 57.30; H, 6.10; N, 8.53.

#### N-allyl-3-benzyl-4-oxo-2-thioxothiazolidine-5-carboxamide (6 g)

Yellow solid; Yield: 75%; MP = 163–165 °C; IR (KBr, v_max_) 3350 (NH), 3028 (C-H Aromatic), 2986 (CH_2_ Aliphatic), 1680 (C=O) Cm^−1^; ^1^H NMR (400 MHz, DMSO-*d*_*6*_) δ 8.31 (t, ^*3*^*J*_*H,H*_ = 5.70 Hz, 1H, NH-CH_2_), 7.35–7.24 (m, 5H, Ph), 5.84–5.33 (m, 1H, CH = CH_2_), 5.15 (d, ^*3*^*J*_*H,H*_ = 15.3 Hz, 2H, CH_2_ = CH), 5.09–4.99 (m, 2H, CH_2_-Ph), 4.84 (dd, ^*3*^*J*_*H,H*_ = 16.6, ^*2*^*J*_*H,H*=_ 3.9 Hz, 1H, CH_Chiral_-CH_2_), 3.71 (t, ^*3*^*J*_*H,H*_ = 5.5 Hz, 2H, CH_2_-NH), 3.12 (dd, ^*3*^*J*_*H,H*_ = 16.6, ^*2*^*J*_*H,H*_ = 8.7 Hz, 1H, H_A__diastropic_, CH_2_-CH_Chiral_), 2.97 (dd, ^*3*^*J*_*H,H*_ = 16.6, ^*2*^*J*_*H,H*_ = 3.9 Hz, 1H, H_B diastropic_, CH_2_-CH_Chiral_), ppm. ^13^C NMR (101 MHz, DMSO-*d*_*6*_): δ 203.45, 176.95, 168.70, 139.46, 135.56, 135.36, 128.85, 127.93, 127.37, 115.84, 47.49, 47.05, 42.72, 41.46, 36.78 ppm; Anal. Calcd: C_14_H_14_N_2_O_2_S_2_: C, 54.88; H, 4.61; N, 9.14; Found C, 55.06; H, 4.58; N, 9.30.

#### 2-(3-benzyl-4-oxo-2-thioxothiazolidin-5-yl)-N-cyclopentylacetamide (6 h)

Brown solid; Yield: 85%; MP = 167–169 °C; IR (KBr, v_max_) 3370(NH), 3055 (C–H Aromatic), 2960 (CH_2_ Aliphatic), 1680 (C=O) Cm^−1^; ^1^H NMR (400 MHz, DMSO-*d*_*6*_) δ 8.10 (d, ^3^*J*_*H,H*_ = 7.3 Hz, 1H, NH), 7.35–7.23 (m, 5H, Ph), 5.15 (d, ^3^*J*_*H,H*_ = 15.1 Hz, 1H, H_A__Diastereotopic_ CH_2_-Ph), 5.04 (d, ^3^*J*_*H,H*_ = 15.1 Hz, 1H, H_B__Diastereotopic_ CH_2_-Ph), 4.81 (dd, ^3^*J*_*H,H*_ = *8.9,* ^2^*J*_*H,H*_ = *3.8* Hz, 1H, CH_Chiral_-CH_2_), 3.98 (d, ^3^*J*_*H,H*_ = 6.7 Hz, 1H, CH_cyclopentyl_-NH), 3.05 (dd, ^3^*J*_H,H_ = 16.5, ^2^*J*_*H,H*_ = 8.7 Hz, 1H, H_A__Diastereotopic_, CH_2_-CH_Chial_), 2.98 (dd, ^*3*^*J*_*H,H*_ = 16.5 Hz, ^2^*J*_*H,H*_ = 8.7 Hz,1H, H_B Diastereotopic_, CH_2_-CH_Chial_), 1.85–1.29 (m, 8H, CH_2cylcopentyl_) ppm. ^13^C NMR (101 MHz, DMSO-*d*_*6*_): δ 203.73, 176.96, 176.95, 168.91, 168.12, 139.46, 135.58, 128.85, 128.00, 127.93, 127.88, 127.76, 127.36 50.89, 47.64, 47.12, 42.73, 36.94, 32.73, 23.87 ppm; Anal. Calcd: C_17_H_20_N_2_O_2_S_2_: C, 58.59; H, 5.78; N, 8.04; Found C, 58.80; H, 5.95; N, 8.24.

#### N-benzyl-3-butyl-4-oxo-2-thioxothiazolidine-5-carboxamide (6i)

Cream solid;Yield:92%; MP = 172–174 °C IR (KBr, v_max_) 3330 (NH), 3025 (C-H Aromatic), 2980 (CH_2_ Aliphatic), 1670 (C=O) cm^−1^; ^1^H NMR (400 MHz, DMSO-d_6_) δ 8.62 (t, ^*3*^*J*_*H,H*_ = 6.00, 1H, NH-CH2), 7.36–7.21 (m, 5H, Ph), 4.72 (dd, ^*3*^*J*_*H,H*_ = *8.40, *^*3*^*J*_*H,H*_ = 3*.*90 Hz, 1H, CH_Chiral_-CH_2_), 4.28 (d, ^*3*^*J*_*H,H*_ = 5.80 Hz, 2H, CH_2_-NH), 3.88 (m, 2H, CH_2__(1)Butyl_), 3.12 (dd, ^*3*^*J*_*H,H*_ = 16.60, ^*2*^*J*_*H,H*_ = 8.40 Hz,1H, H_A__diastropic_, CH_2_-CH_Chiral_), 2.98 (dd, ^*3*^*J*_*H,H*_ = 16.60, ^*3*^*J*_*H,H*_ = 8.40 Hz ,1H, H_B__diastropic_, CH_2_–CH_Chiral_), 1.55 (p, ^*3*^*J*_*H,H*_ = 7.50 Hz, 2H, CH_2__(2_) _Butyl_), 1.30 (s, ^*3*^*J*_*H,H*_ = 7.50 Hz, 2H, CH_2__(3)__Butyl_), 0.89 (t, ^*3*^*J*_*H,H*_ = 7.30 Hz, 3H, CH_3_). ppm. ^13^C NMR (101 MHz, DMSO-*d*_*6*_): δ 203.61, 176.83, 168.90, 139.47, 128.77, 127.72, 127.34, 46.77, 44.26, 42.70, 36.92, 28.73, 19.94, 14.08 ppm; ESI–MS (C_16_H_20_N_2_O_2_S_2_): calculated m/z 336.10 [M + H]^+^, observed m/z 336.19 [M + H]^+^; Anal. Calcd : C_16_H_20_N_2_O_2_S_2_; C, 57.11; H, 5.99; N, 8.33; Found C, 57.30; H, 6.10; N, 8.55.

#### 2-(3-butyl-4-oxo-2-thioxothiazolidin-5-yl)-N-(4-methylbenzyl)acetamide (6j)

Cream solid; Yield: 88%; MP = 174–176 °C; IR (KBr, v_max_) 3360 (NH), 3039 (C-H Aromatic), 2967 (CH_2_ Aliphatic), 1685 (C=O) cm^−1^; ^1^H NMR (400 MHz, DMSO-*d*_*6*_) δ 8.56 (t, ^*3*^*J*_*H,H*_ = 5.90 Hz, 1H, NH-CH_2_), 7.13 (s, 5H, Ph), 4.71 (dd, ^*3*^*J*_*H,H*_ = 8.40, ^*2*^*J*_*H,H*_ = 3.90 Hz, 1H, CH_chairal_-CH_2_), 4.22 (d, ^*3*^*J*_*H,H*_ = 5.90 Hz, 2H, CH_2_-CH_chairal_), 3.93–3.80 (m, 2H, CH_2__(1)__butyl_), 3.10 (dd, ^*3*^*J*_*H,H*_ = 16.60, ^*2*^*J*_*H,H*_ = 8.20 Hz, 1H, H_A__diastropic_, CH_2_-CH_Chiral_), 2.96 (dd, ^*3*^*J*_*H,H*_ = 16.60, ^*2*^*J*_*H,H*_ = 8.20 Hz, 1H, H_B__diastropic_, CH_2_-CH _Chiral_), 2.28 (s, 3H, CH_3_-Ph), 1.59–1.53 (m, 2H, CH_2__(2)butyl_), 1.35–1.24 (m, 2H, CH_2__(3)butyl_), 0.89 (t, ^*3*^*J*_*H,H*_ = 7.30 Hz, 3H, CH_3__Butyl_) ppm. ^13^C NMR (101 MHz, DMSO-*d*_*6*_): δ 203.63, 176.85, 168.80, 136.42, 136.40, 129.31, 127.72, 46.77, 44.24, 42.44, 36.91, 28.72, 21.14, 19.93, 14.08 ppm; Anal. Calcd: C_17_H_22_N_2_O_2_S_2_: C, 58.25; H, 6.33; N, 7.99; Found C, 58.45; H, 6.55; N, 8.19.

#### 2-(3-butyl-4-oxo-2-thioxothiazolidin-5-yl)-N-isobutylacetamide (6 k)

Cream solid; Yield: 79%; MP = 170–172 °C; IR (KBr, v_max_) 3335 (NH), 2944 (CH_2_ Aliphatic), 1660 (C=O) cm^-1^; ^1^H NMR (400 MHz, DMSO-*d*_*6*_) δ 8.07 (t, ^*3*^*J*_*H,H*_ = 5.00 Hz, 1H, NH-CH_2_), 4.67 (dd, ^*3*^*J*_*H,H*_ = 8.60, ^*3*^*J*_*H,H*_ = 3.90, Hz, 1H, CH_Chairal_-CH_2_), 3.86 (q, ^*3*^*J*_*H,H*_ = 6.80 Hz, 2H, CH_2__Isobutyl_-NH), 3.04 (dd, ^*3*^*J*_*H,H*_ = 12.50, ^*2*^*J*_*H,H*_ = 8.40 Hz, 1H, H_A__diastropic_, CH_2_-CH _Chiral_), 2.93–2.81 (m, 3H, H_B__diastropic_, CH_2_-CH _Chiral_, CH_2__(1)__Butyl_), 1.65 (d, t, ^*3*^*J*_*H,H*_ = 13.40, ^*2*^*J*_*H,H*_ = 6.70 Hz, 1H, CH_Isobutyl_), 1.54 (p, ^*3*^*J*_*H,H*_ = 7.50 Hz, 2H, CH_2 (2) Butyl_), 1.30 (s, ^*3*^*J*_*H,H*_ = 7.40 Hz 2H, CH_2__(3)__Butyl_), 0.89 (t, ^*3*^*J*_*H,H*_ = 7.30 Hz, 3H, CH_3__Butyl_), 0.83 (d, ^*3*^*J*_*H,H*_ = 6.70 Hz, 6H, 2 × CH_3__Isobutyl_) ppm. ^13^C NMR (101 MHz, DMSO-*d*_*6*_): δ 203.64, 176.84, 168.71, 46.81, 46.62, 44.21, 38.77, 36.95, 31.56, 28.72, 28.50, 20.52, 19.93, 14.04 ppm; Anal. Calcd: C_13_H_22_N_2_O_2_S_2_: C, 51.62; H, 7.33; N, 9.26; Found C, 51.80; H, 7.55; N, 9.45.

#### N-benzyl-2-(3-isobutyl-4-oxo-2-thioxothiazolidin-5-yl)acetamide (6 l)

Brown solid; Yield: 75%; MP = 169–171 °C; IR (KBr, v_max_) 3356(NH), 3020 (C-H Aromatic), 2935 (CH_2_ Aliphatic), 1655(C=O) Cm^−1^; ^1^H NMR (400 MHz,DMSO-*d*_*6*_) δ 8.86 (t, ^*3*^*J*_*H,H*_ = 6.00 Hz, 1H, NH-CH_2_), 7.40–7.21 (m, 5H, Ph), 4.77 (dd, ^*3*^*J*_*H,H*_ = 8.50, ^*2*^*J*_*H,H*_ = 3.90, Hz, 1H, CH_chairal_-CH_2_), 4.28 (d, ^*3*^*J*_*H,H*_ = 5.90 Hz, 2H, CH_2_-NH), 3.80–3.65 (m, CH_2__Isobutyl_), 3.13 (dd, ^*3*^*J*_*H,H*_ = 16.60, ^*2*^*J*_*H,H*_ = 8.60 Hz, 1H, H_A__diastropic_, CH_2_-CH_Chiral_), 2.98 (dd, ^*3*^*J*_*H,H*_ = 16.60, ^*2*^*J*_*H,H*_ = 8.60 Hz, 1H, H_B__diastropic_, CH_2_-CH_Chiral_), 2.17 (t, ^*3*^*J*_*H,H*_ = 13.8, 6.90 Hz, 1H, CH_Isobutyl_), 0.87 (d, ^*3*^*J*_*H,H*_ = 6.70 Hz, 6H, 2 × CH_3__Isobutyl_) ppm. ^13^C NMR (101 MHz, DMSO-*d*_*6*_): δ 204.45, 177.81, 168.90, 139.46, 128.78, 127.73, 127.35, 51.42, 46.65, 42.69, 36.93, 26.73, 20.47, 20.42, ppm; Anal. Calcd : C_16_H_20_N_2_O_2_S_2_: C, 57.11; H, 5.99; N, 8.33; Found C, 57.31; H, 6.15; N, 8.55.

#### 2-(3-isobutyl-4-oxo-2-thioxothiazolidin-5-yl)-N-(4-methylbenzyl)acetamide (6 m)

Yellow solid; Yield: 82%; MP = 173–175 °C; IR (KBr, v_max_) 3365(NH), 3045 (C-H Aromatic), 2954 (CH_2_ Aliphatic), 1660 (C=O) Cm^−1^; ^1^H NMR (400 MHz, DMSO-*d*_*6*_) δ 8.57 (t, ^*3*^*J*_*H,H*_ = 6.00 Hz, 1H, NH-CH_2_), 7.13 (s, 4H, Ph), 4.75 (dd, ^*3*^*J*_*H,H*_ = 8.70, ^*2*^*J*_*H,H*_ = 3.90, Hz, 1H, CH_chairal_-CH_2_), 4.22 (d, ^*3*^*J*_*H,H*_ = 5.70 Hz, 2H, CH_2_-NH), 3.78–3.64 (m, 2H, CH_2__Isopropyl_), 3.11 (dd, ^*3*^*J*_*H,H*_ = 16.50, ^*2*^*J*_*H,H*_ = 8.60 Hz, 1H, H_A__diastropic_, CH_2_-CH_Chiral_), 2.96 (dd, ^*3*^*J*_*H,H*_ = 16.50, ^*2*^*J*_*H,H*_ = 8.60 Hz, 1H, H_B__diastropic_, CH_2_-CH_Chiral_), 2.28 (s, 3H, CH_3_-Ph), 2.21–2.04 (m, 1H, CH_Isopropyl_), 0.87 (d, ^*3*^*J*_*H,H*_ = 6.90, 6H, 2CH_3__Isobutyl_) ppm. ^13^C NMR (101 MHz, DMSO-*d*_*6*_): δ 204.15, 177.52, 168.50, 136.41, 129.31, 127.73, 51.42, 46.65, 42.44, 36.63, 26.73, 21.14, 20.45, 20.42 ppm; Anal. Calcd: C_17_H_22_N_2_O_2_S_2_: C, 58.25; H, 6.33; N, 7.99; Found C, 58.45; H, 6.53; N, 8.20.

#### 2-(3-isobutyl-4-oxo-2-thioxothiazolidin-5-yl)-N-propylacetamide (6n)

Brown solid; Yield: 84%; MP = 160–162 °C; IR (KBr, v_max_) 3329(NH), 2940 (CH_2_ Aliphatic), 1645 (C=O) Cm^−1^; ^1^H NMR (400 MHz, DMSO-*d*_*6*_) δ 8.08 (t, ^*3*^*J*_*H,H*_ = 5.70 Hz, 1H, NH-CH_2_), 4.71 (dd, ^*3*^*J*_*H,H*_ = 8.70, ^*2*^*J*_*H,H*_ = 3.80, Hz, 1H, CH_chairal_-CH_2_), 3.71 (d,d ^*3*^*J*_*H,H*_ = 13.00, ^*2*^*J*_*H,H*_ = 7.40 Hz, 2H, CH_2__Propyl_-NH), 3.12–2.97 (m, 3H, H_A__diastropic_, CH_2_-CH_Chiral_, CH_2 Propyl_), 2.87 (dd, ^*3*^*J*_*H,H*_ = 16.50, ^*2*^*J*_*H,H*_ = 8.70 Hz, 1H, H_B__diastropic_, CH_2_-CH _Chiral_), 2.20–2.13 (m, 1H, CH_Isobutyl_), 1.39 (q, ^*3*^*J*_*H,H*_ = 7.2 Hz, 2H, CH_2__Isobutyl_), 0.90–0.80 (m, 9H, 2 × CH_3__Isobutyl_, CH_3 Propyl_) ppm. ^13^C NMR (101 MHz, DMSO-*d*_*6*_): δ 204.20, 177.22, 168.60, 51.30, 46.66, 40.91, 36.39, 26.72, 22.74, 20.45, 20.40, 11.83 ppm; Anal. Calcd: C_12_H_20_N_2_O_2_S_2_: C, 49.97; H, 6.99; N, 9.71; Found C, 50.15; H, 7.19; N, 9.90.

#### N-allyl-2-(3-isobutyl-4-oxo-2-thioxothiazolidin-5-yl)acetamide (6o)

Cream solid; Yield: 77%; MP = 164–166 °C; IR (KBr, v_max_) 3338 (NH), 2952 (CH_2_ Aliphatic), 1665(C=O) Cm^−1^; ^1^H NMR (400 MHz, DMSO-*d*_*6*_) δ 8.27 (t, ^*3*^*J*_*H,H*_ = 5.70 Hz, 1H, NH-CH_2_), 5.84–5.72 (m, 1H, CH _Allyl_), 5.15 (d, ^*3*^*J*_*H,H*_ = 17.10 Hz, 1H, H_A_, CH_2__Allyl_), 5.07 (d, ^*3*^*J*_*H,H*_ = 12.00 Hz, 1H, H_B_, CH_2__Allyl_), 4.72 (dd, ^*3*^*J*_*H,H*_ = 8.60, ^*2*^*J*_*H,H*_ = 3.90 Hz, 1H, CH_2__Chiral_-CH), 3.80–3.64 (m, 4H, CH_2_-NH, CH_2__Isobutyl_), 3.08 (dd, ^*3*^*J*_*H,H*_ = 16.60, ^*3*^*J*_*H,H*_ = 8.70 Hz, 1H, H_A__diastropic_, CH_2_-CH_Chiral_), 2.92 (dd, ^*3*^*J*_*H,H*_ = 16.60, ^*3*^*J*_*H,H*_ = 8.70 Hz, 1H, H_B diastropic_, CH_2_-CH_Chiral_), 2.16 (d t, ^*3*^*J*_*H,H*_ = 13.80, ^*2*^*J*_*H,H*_ = 6.90 Hz, 1H, CH_Isoutyl_), 0.87 (d, ^*3*^*J*_*H,H*_ = 6.70 Hz, 6H, 2CH_3__Isobutyl_) ppm. ^13^C NMR (101 MHz, DMSO-*d*_*6*_): δ 204.75, 177.70, 168.66, 135.36, 135.76, 51.40, 46.60, 41.44, 36.84, 26.72, 20.45, 20.41 ppm; Anal. Calcd: C_12_H_18_N_2_O_2_S_2_: C, 50.32; H, 6.33; N, 9.78; Found C, 50.55; H, 6.53; N, 9.98.

### In vitro urease inhibition assay

The assay was performed exactly according to our previous report. Briefly, 850 μL of urea and 15 μL urease (0.135 units dissolved in PBS, pH 7.4) were added to 100 μL of the synthesized derivatives at different concentrations. After 30 min, to 100 μL of the incubated solution, 500 μL solution I (5.0 g phenol and 25.0 mg sodium nitroprusside in 500 mL water) was added followed by the addition of 500 μL of solution II (2.5 g sodium hydroxide, 4.2 mL sodium hypochlorite, and 5% chlorine in 500 mL water) which was further incubated at 37 °C for 30 min. The absorbance was determined by measuring indophenols at 625 nm. Thiourea was used as the standard inhibitor for urease. The IC_50_ values for all synthesized compounds were calculated using GraphPad Prism software (GraphPad Software, Inc., San Diego, CA)^20^.

### Enzyme kinetic studies

The mode of inhibition of the most active compound (6i), identified with the lowest IC_50_, was investigated against urease activity with different concentrations of urea as substrate in the absence and presence of 6i at different concentrations (0, 0.5, 1, and 2 µM). A Lineweaver–Burk plot was generated to identify the type of inhibition, and the Michaelis–Menten constant (*K*_m_) value was determined from the plot between the reciprocal of the substrate concentration (1/[S]) and reciprocal of enzyme rate (1/V) over various inhibitor concentrations. The experimental inhibitor constant (*K*_i_) value was constructed by secondary plots of the inhibitor concentration [I] versus *K*_m_.

### In silico studies

Maestro molecular modeling platform (version 10.5) by Schrödinger, LLC (Maestro, Schrödinger, LLC, New York, NY, 2021) was used to perform the docking study of compound 6i and urea on the urease enzyme. X-ray crystallographic structure of jack bean urease was downloaded from the protein data bank (www.rcsb.com) by the PDB ID: 4h9m. All the water molecules and co-crystallized ligands were removed using a protein preparation wizard^24^. Afterward missing sidechains and loops were filled using the prime tool^25^. The 2D structure of ligands was drawn in ChemDraw (ver. 16) and saved as SDF files. The Ligprep module^26^ was used to prepare ligand molecules with an OPLS_2005 force field and using EPIK^27^ at a target pH of 7.0 ± 2. Docking simulation was conducted using IFD^28^ in which the AHA choose the grid center with the maximum number of 20 poses for each ligand. Receptor and ligand van der Waals radii have been set as 0.7 and 0.5 Å respectively. Structures with prime energy levels beyond 30 kcal/mol were eliminated based on standard precious glide docking. The molecular dynamic simulation was performed using Desmond^29^. Proper complex for MD simulation obtained from the IFD results. The simulation was conducted in an orthorhombic cell filled with TI3P model water molecules and adequate Na ions to neutralize the overall charge of the complex. The NPT ensemble (constant number of atoms; constant pressure, i.e., 1.01325 bar; and constant temperature, i.e., 300 K) was used considering The 1.0‐ps interval Nose–Hoover chain method as the default thermostat with and 2.0‐ps intervals Martyna–Tobias–Klein as the default barostat. The overall duration of simulation has been set on 20 ns for both urease-thiourea and urease-compound 6i complexes. For each 100 ps one trajectory was stored. The results of molecular dynamic simulation have been analyzed using the maestro graphical interface.

### Urease inhibitory activity

To assess the inhibition of urease activity by synthetic compounds, Christensen’s urea.

medium containing urea and phenol red (pH indicator) was used for the preparation of two-fold serial dilutions of compounds in 96-well microtiter plates. Cell suspension of a urease-positive clinical isolate of *P. vulgaris* was prepared in a urea medium and added to each well. The turbidity of bacterial suspension was adjusted to 0.5 McFarland using a spectrophotometer, and also, the density of inoculated suspension was 1 × 10^6^ CFU/ml. The concentrations of compounds were in the range of 1–512 µg/ml.

Inoculated media without adding any compound was considered a positive control, while uninoculated media were used as a negative control (blank). The anti-urease activity was assessed spectroscopically at 560 nm after 24 h incubation at 37 °C^14^.

### Antimicrobial activity

The antimicrobial activity of designed compounds was investigated using broth microdilution.

method according to procedures suggested by the Clinical & Laboratory Standards Institute (CLSI) against standard/clinical strains of microorganisms including *S. aureus* (ATCC 25923), *E. coli* (ATCC 25922), a clinical isolate of *P. vulgaris*, and *C. albicans* (ATCC 10261). Briefly, twofold serial dilutions of the compounds were prepared in Mueller–Hinton Broth (MHB, HiMedia) for bacterial strains and RPMI-1640 (Sigma-Aldrich) for yeast strain (Ranging 1–512 µg/ml). Then, 100 μL of each dilution was transferred into the well of a microtiter plate and inoculated with 100 μL of cell suspension with a final density of 1–5 × 10^3^ CFU/ml for yeasts and 1–1.5 × 10^6^ cells/ml for bacteria. Plates were incubated at proper temperature overnight, and the minimum inhibitory concentration (MIC) of compounds was determined as the lowest concentration that prevented visible growth of tested microorganisms compared with the growth in the control wells (wells without tested compounds). Moreover, the minimum bactericidal/fungicidal concentration (MBC/MFC) of compounds was determined by transferring the 10 µl of clear wells into the agar media. Standard antibacterial and antifungal agents (Ciprofloxacin and Fluconazole) were evaluated. All experiments were performed in duplicate^30,31^.

### Inhibition of biofilm formation

To evaluate the ability of the synthesized compounds **6a-i** to prevent biofilm formation by standard species of *S. aureus* (ATCC25923) and MRSA strain, biofilms were pre-formed in 96 well plates, as previously described^32^. Firstly, bacterial strains were inoculated into Tryptic Soy Broth (TSB) medium. The cells were cultivated on a shaker overnight for 180 r/min at 37 °C. Then, the suspension was centrifuged, and the cells were washed with PBS three times. Diluted cell suspension of strains was added into the wells with 1 × 10^6^ final concentration. The plates were incubated for 4 h at 37 °C. After that, 100 μl of each of the dissolved compounds was added to a final concentration of 512 mg/ml in the wells. The plates were further incubated for 24 h at 37 °C. Following incubation, the crystal violet assay was performed to evaluate the inhibition of biofilm formation. For this purpose, the supernatant of the wells was aspirated gently, and the wells were washed three times with sterile distilled water. After completely dried, the wells were stained with 100 μl of 1% crystal violet and incubated at room temperature for 15 min. After staining, the plates were washed three times with sterile distilled water to remove the extra stain. Then, 130 μl of 95% ethanol was added to destain the wells. Assessment of biofilm formation was performed by transferring 100 μl of the destaining solution to a new plate and measuring the absorbance at 590 nm using a microplate reader. The assay was repeated at least three times^33,34^. The mean absorbance (OD_590_ _nm_) of the samples was determined, and percentage inhibition was obtained using Eq. (1) as follows:

1 $${\text{Percentage }}\;{\text{inhibition}}:\;{\text{ OD }}\;{\text{blank }}{-}{\text{ OD }}\;{\text{test }} \times { 1}00/{\text{OD}}\;{\text{ blank}}$$

### In vitro cytotoxic evaluation

MOLT-4 (human acute lymphoblastic leukemia) cells were cultures in RPMI-1640 medium supplemented with 10% heat-inactivated fetal bovine serum. Cell viability was measured by the MTT assay. MOLT-4 cells were seeded into 96- well micro-culture plates. After 24 h, the culture medium was replaced with a medium containing different concentrations of newly synthesized derivatives. Control wells were supplemented with the same volume of growth medium not containing any drugs. Cells were then incubated at 37 °C for 72 h. At the end of the exposure time, the medium was removed and MTT solution of 0.5 mg/ml was added to each well. The plates were incubated at 37 °C for 4 h, after which DMSO was added to each well to solubilize the formed formazan crystals, and the absorbance of each well was read with a microplate reader at 570 nm.

## Supplementary Information

Supplementary Information.

## Data Availability

All data generated and/or analyzed during this study are included in this article. All data generated or analyzed during this study are included in the supplementary information file.
