# Supplementary material for: New thioxothiazolidinyl-acetamides derivatives as potent urease inhibitors: design, synthesis, in vitro inhibition, and molecular dynamic simulation
Source: Sci Rep. 2023 Jan 2;13:21. doi: 10.1038/s41598-022-27234-3 (PMC9807592; doi:10.1038/s41598-022-27234-3)
Supplement: Supplementary file 1 — Supplementary Information. [file 41598_2022_27234_MOESM1_ESM.docx]

**Supporting Information**

**New thioxothiazolidinyl-acetamides derivatives as potent urease inhibitors; Design, synthesis, in vitro inhibition, and molecular dynamic simulation**

Navid Dastyafteh^1^, Milad Noori^1^, Mohammad Nazari Montazer^2^, Kamiar Zomorodian^3^, Somayeh Yazdanpanah^3^, Aida Iraji^4,5^, Minoo Khalili Ghomi^1^, Shahrzad Javanshir^6^, Mehdi Asadi^2^, Mehdi Dianatpour^3^, Mahmood Biglar^7^, Bagher Larijani^1^, Massoud Amanlou^2,7*^, Mohammad Mahdavi^1*^

1. Endocrinology and Metabolism Research Center, Endocrinology and Metabolism Clinical Sciences Institute, Tehran University of Medical Sciences, Tehran, Iran.

2. Department of Medicinal Chemistry, Faculty of Pharmacy, Tehran University of Medical Sciences, Tehran, Iran.

3. Department of Medical Mycology and Parasitology, School of Medicine, Shiraz University of Medical Sciences, Shiraz, Iran

4. Stem Cells Technology Research Center, Shiraz University of Medical Sciences, Shiraz, Iran

5. Central Research Laboratory, Shiraz University of Medical Sciences, Shiraz, Iran

6. Department of Chemistry, Iran University of Science and Technology, Tehran, Iran

7. Drug Design and Development Research Center, The Institute of Pharmaceutical Sciences (TIPS), Tehran University of Medical Sciences, Tehran, Iran.

Contents Pages

HNMR Spectrum of N-benzyl-2-(3-benzyl-4-oxo-2-thioxothiazolidin-5-yl)acetamide (6a) 3

CNMR Spectrum of N-benzyl-2-(3-benzyl-4-oxo-2-thioxothiazolidin-5-yl)acetamide (6a) 3

HNMR Spectrum of 2-(3-benzyl-4-oxo-2-thioxothiazolidin-5-yl)-N-(4-methylbenzyl)acetamide) (6b) 4

CNMR Spectrum of 2-(3-benzyl-4-oxo-2-thioxothiazolidin-5-yl)-N-(4-methylbenzyl)acetamide) (6b) 4

HNMR Spectrum of 2-(3-benzyl-4-oxo-2-thioxothiazolidin-5-yl)-N-(4-fluorobenzyl)acetamide (6c) 5

CNMR Spectrum of 2-(3-benzyl-4-oxo-2-thioxothiazolidin-5-yl)-N-(4-fluorobenzyl)acetamide (6c) 5

HNMR Spectrum of 2-(3-benzyl-4-oxo-2-thioxothiazolidin-5-yl)-N-propylacetamide (6d) 6

CNMR Spectrum of 2-(3-benzyl-4-oxo-2-thioxothiazolidin-5-yl)-N-propylacetamide (6d) 6

HNMR Spectrum of 2-(3-benzyl-4-oxo-2-thioxothiazolidin-5-yl)-N-butylacetamide (6e) 7

CNMR Spectrum of 2-(3-benzyl-4-oxo-2-thioxothiazolidin-5-yl)-N-butylacetamide (6e) 7

HNMR Spectrum of 2-(3-benzyl-4-oxo-2-thioxothiazolidin-5-yl)-N-isobutylacetamide (6f) 8

CNMR Spectrum of 2-(3-benzyl-4-oxo-2-thioxothiazolidin-5-yl)-N-isobutylacetamide (6f) 8

HNMR Spectrum of N-allyl-2-(3-benzyl-4-oxo-2-thioxothiazolidin-5-yl)acetamide (6g) 9

CNMR Spectrum of N-allyl-2-(3-benzyl-4-oxo-2-thioxothiazolidin-5-yl)acetamide (6g) 9

HNMR Spectrum of 2-(3-benzyl-4-oxo-2-thioxothiazolidin-5-yl)-N-cyclopentylacetamide (6h) 10

CNMR Spectrum of 2-(3-benzyl-4-oxo-2-thioxothiazolidin-5-yl)-N-cyclopentylacetamide (6h) 10

HNMR Spectrum of N-benzyl-2-(3-butyl-4-oxo-2-thioxothiazolidin-5-yl)acetamide (6i) 11

CNMR Spectrum of N-benzyl-2-(3-butyl-4-oxo-2-thioxothiazolidin-5-yl)acetamide (6i) 11

HNMR Spectrum of 2-(3-butyl-4-oxo-2-thioxothiazolidin-5-yl)-N-(4-methylbenzyl)acetamide (6j) 12

CNMR Spectrum of 2-(3-butyl-4-oxo-2-thioxothiazolidin-5-yl)-N-(4-methylbenzyl)acetamide (6j) 12

HNMR Spectrum of 2-(3-butyl-4-oxo-2-thioxothiazolidin-5-yl)-N-isobutylacetamide (6k) 13

CNMR Spectrum of 2-(3-butyl-4-oxo-2-thioxothiazolidin-5-yl)-N-isobutylacetamide (6k) 13

HNMR Spectrum of N-benzyl-2-(3-isobutyl-4-oxo-2-thioxothiazolidin-5-yl)acetamide (6l) 14

CNMR Spectrum of N-benzyl-2-(3-isobutyl-4-oxo-2-thioxothiazolidin-5-yl)acetamide (6l) 14

HNMR Spectrum of 2-(3-isobutyl-4-oxo-2-thioxothiazolidin-5-yl)-N-(4-methylbenzyl)acetamide (6m) 15

CNMR Spectrum of 2-(3-isobutyl-4-oxo-2-thioxothiazolidin-5-yl)-N-(4-methylbenzyl)acetamide (6m) 15

HNMR Spectrum of 2-(3-isobutyl-4-oxo-2-thioxothiazolidin-5-yl)-N-propylacetamide (6n) 16

CNMR Spectrum of 2-(3-isobutyl-4-oxo-2-thioxothiazolidin-5-yl)-N-propylacetamide (6n) 16

HNMR Spectrum of N-allyl-2-(3-isobutyl-4-oxo-2-thioxothiazolidin-5-yl)acetamide (6o) 17

CNMR Spectrum N-allyl-2-(3-isobutyl-4-oxo-2-thioxothiazolidin-5-yl)acetamide (6o) 17


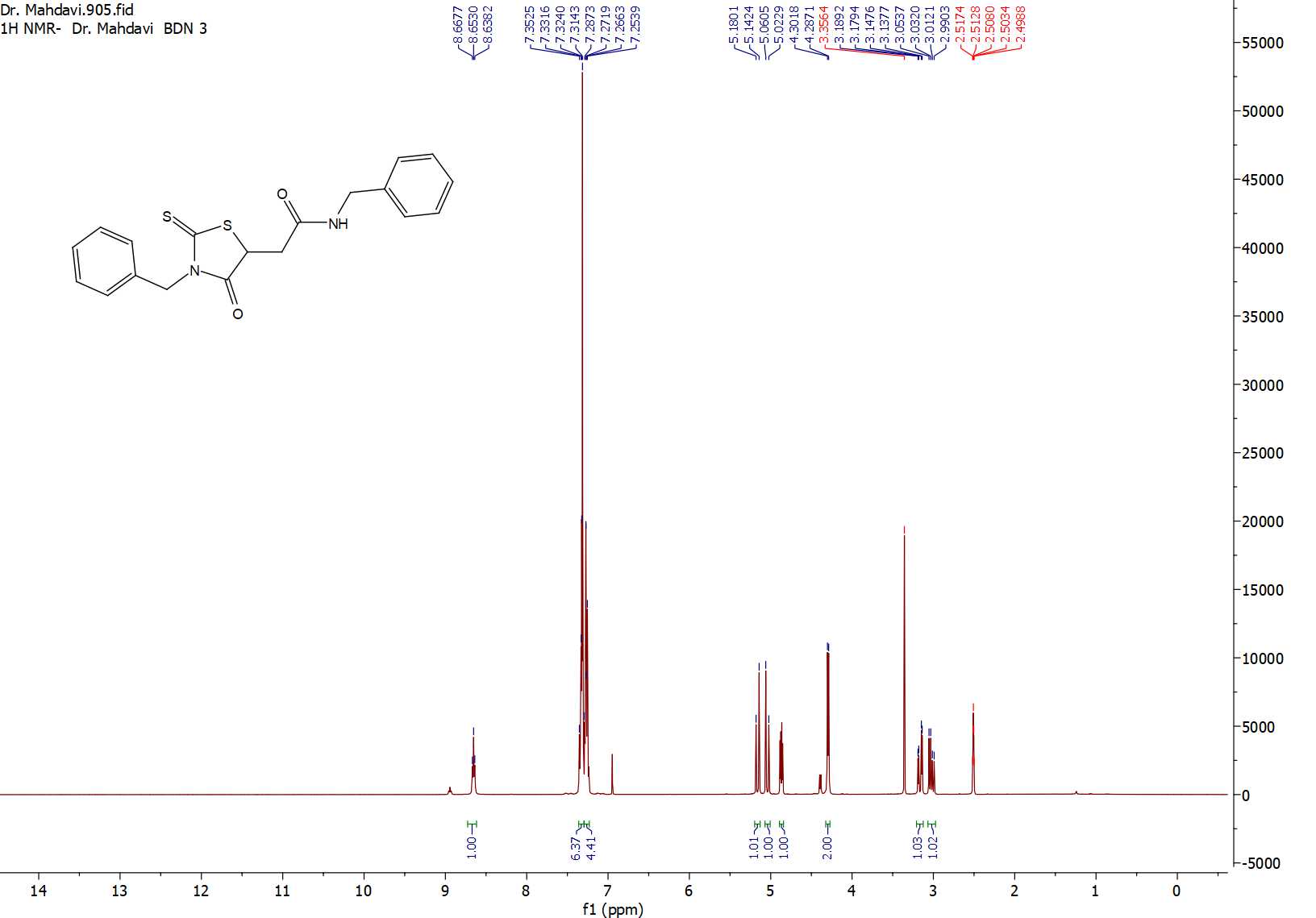


Figure S1. HNMR Spectrum of N-benzyl-2-(3-benzyl-4-oxo-2-thioxothiazolidin-5-yl)acetamide (6a)


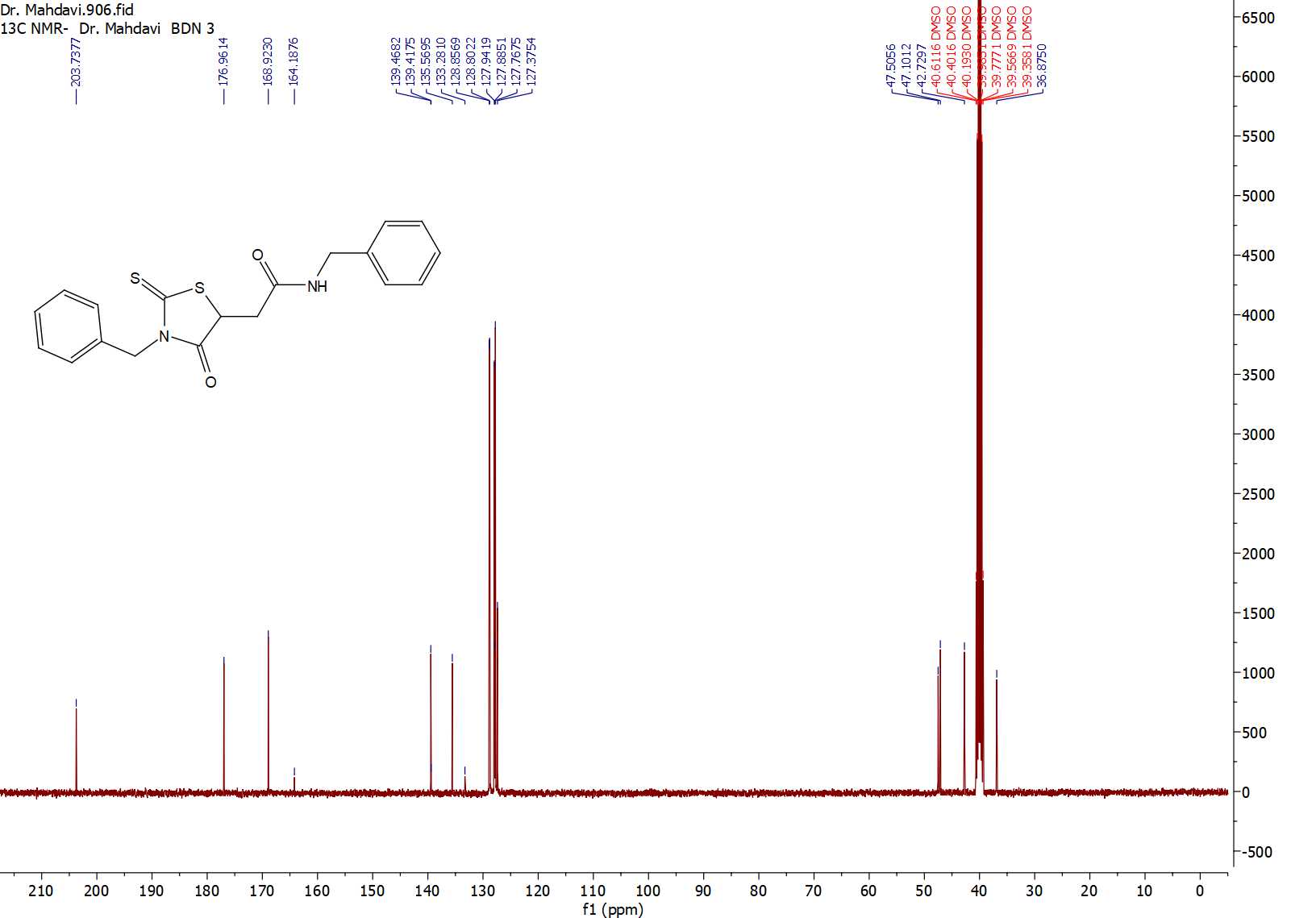


Figure S2. CNMR Spectrum of N-benzyl-2-(3-benzyl-4-oxo-2-thioxothiazolidin-5-yl)acetamide (6a)


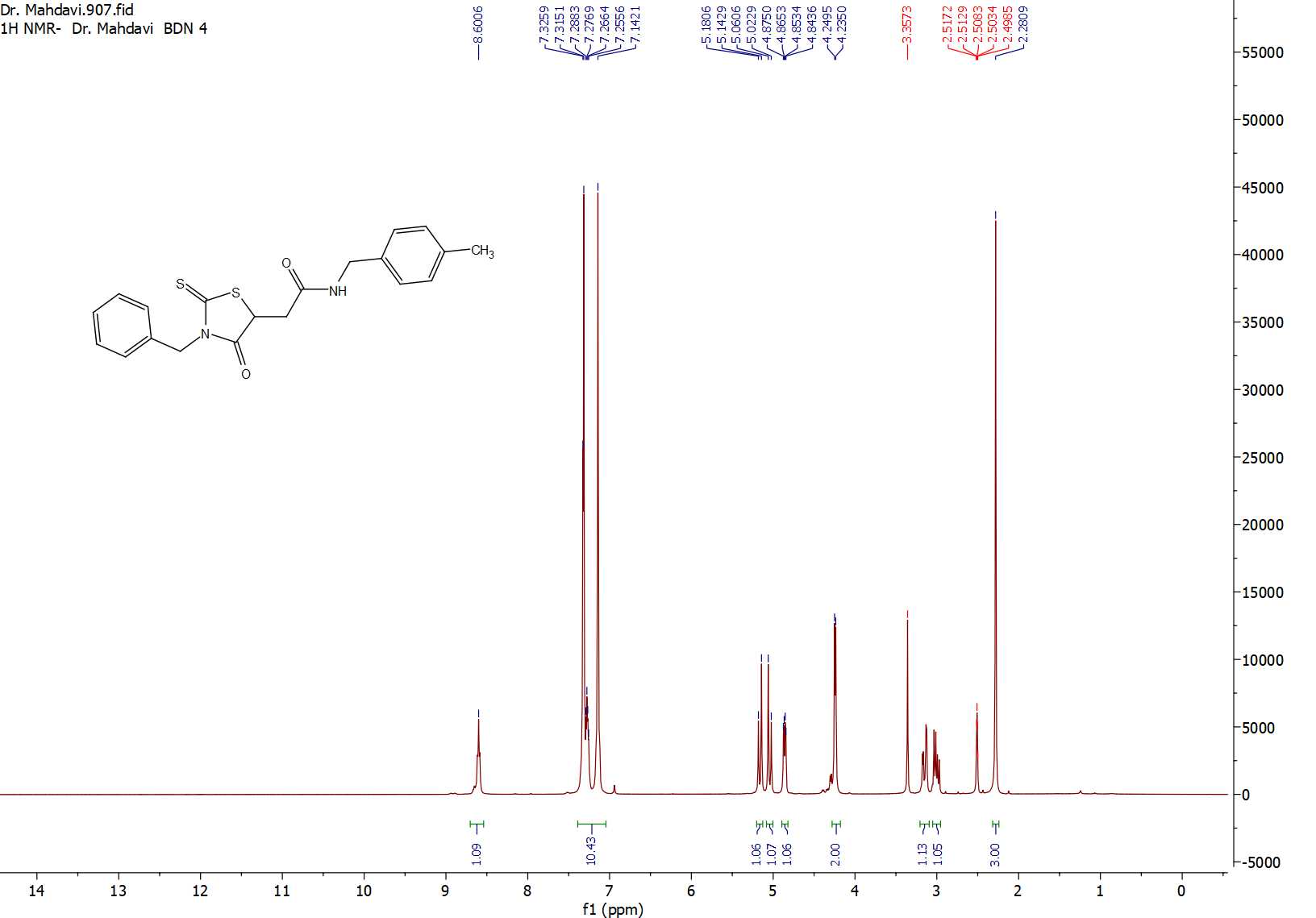


Figure S3. HNMR Spectrum of 2-(3-benzyl-4-oxo-2-thioxothiazolidin-5-yl)-N-(4-methylbenzyl)acetamide) (6b)


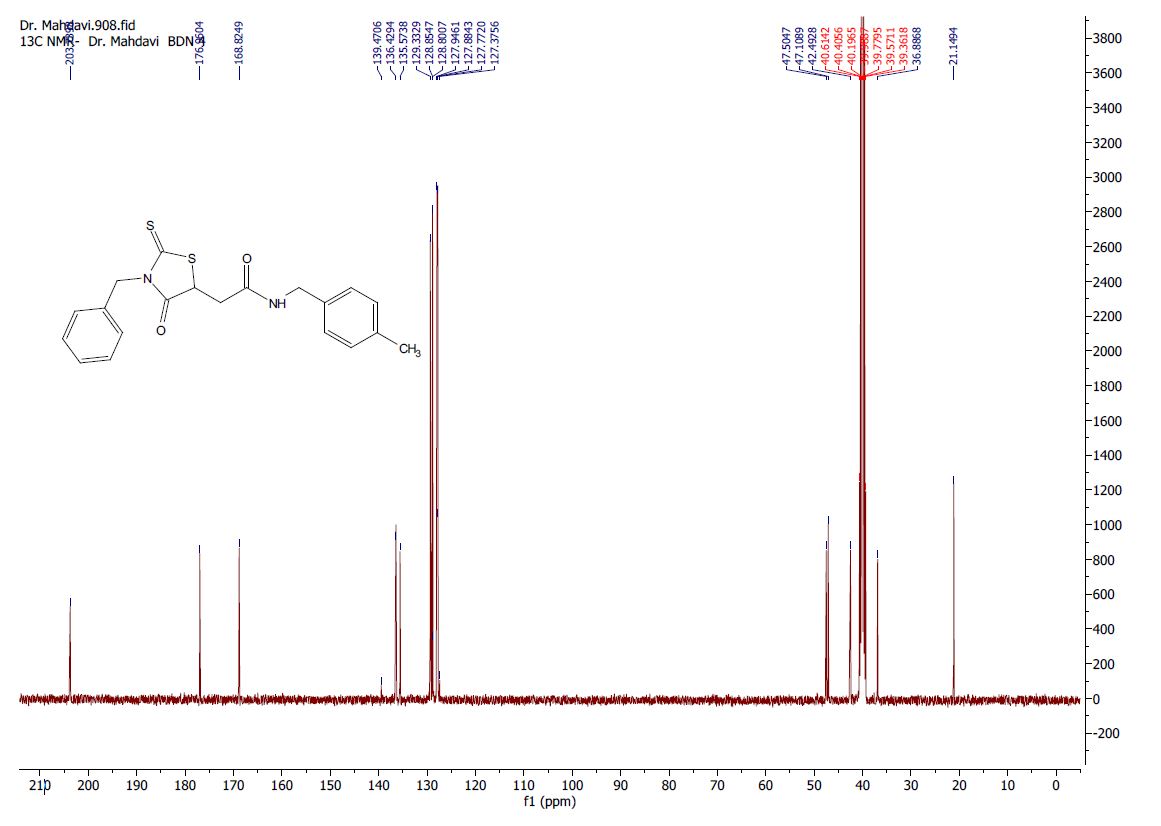


Figure S4. CNMR Spectrum of 2-(3-benzyl-4-oxo-2-thioxothiazolidin-5-yl)-N-(4-methylbenzyl)acetamide) (6b)


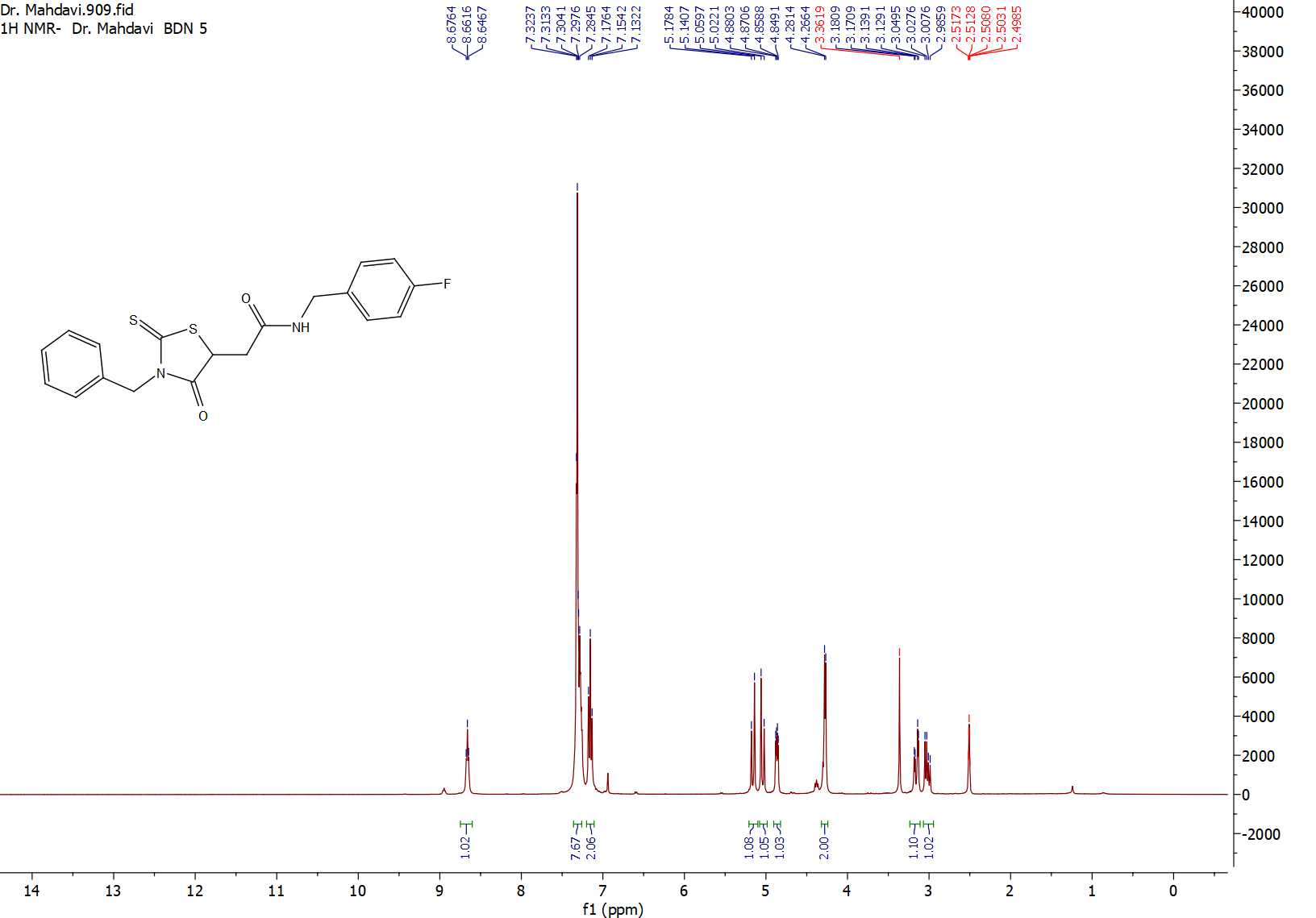


Figure S5. HNMR Spectrum of 2-(3-benzyl-4-oxo-2-thioxothiazolidin-5-yl)-N-(4-fluorobenzyl)acetamide (6c)


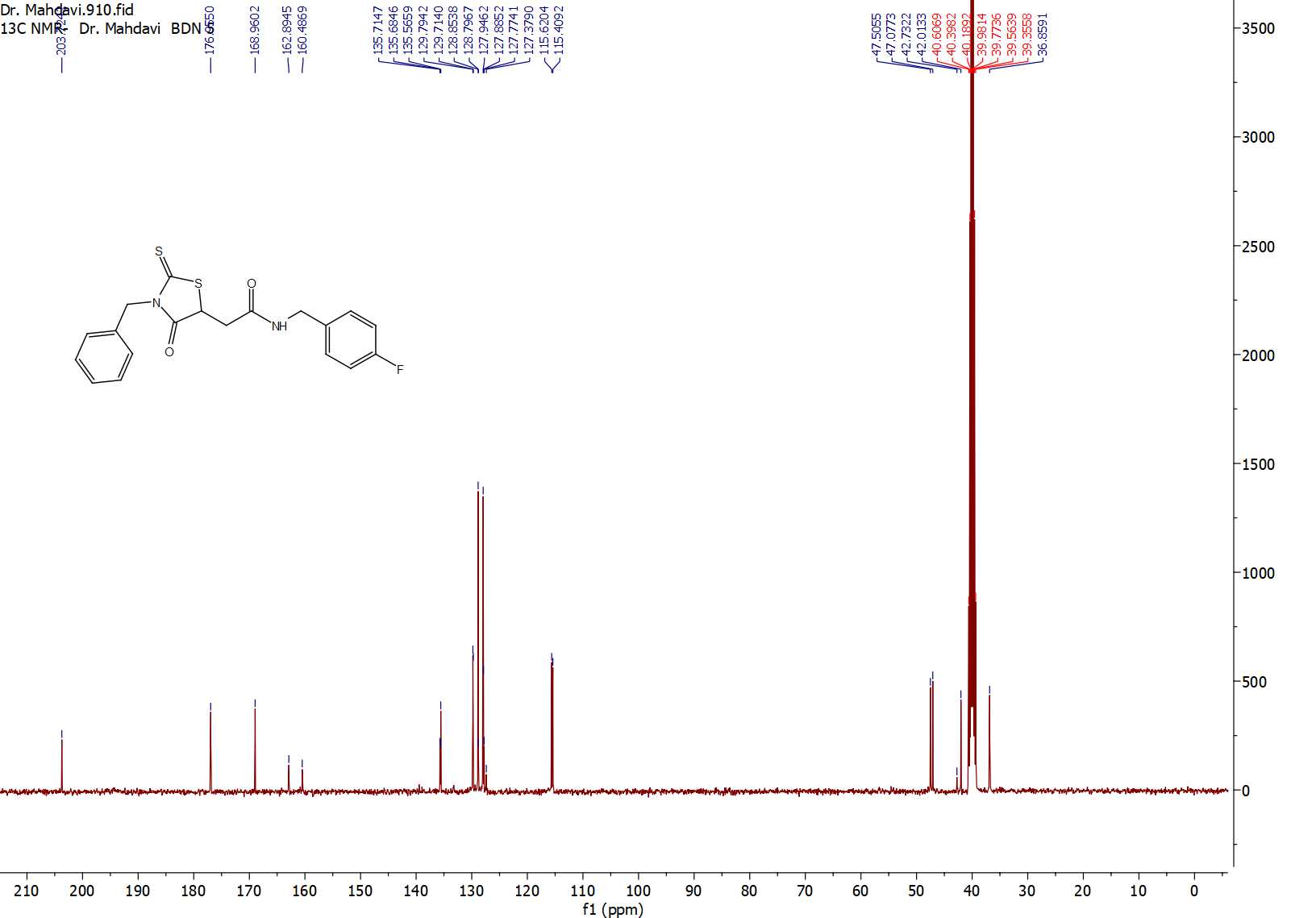


Figure S6. CNMR Spectrum of 2-(3-benzyl-4-oxo-2-thioxothiazolidin-5-yl)-N-(4-fluorobenzyl)acetamide (6c)


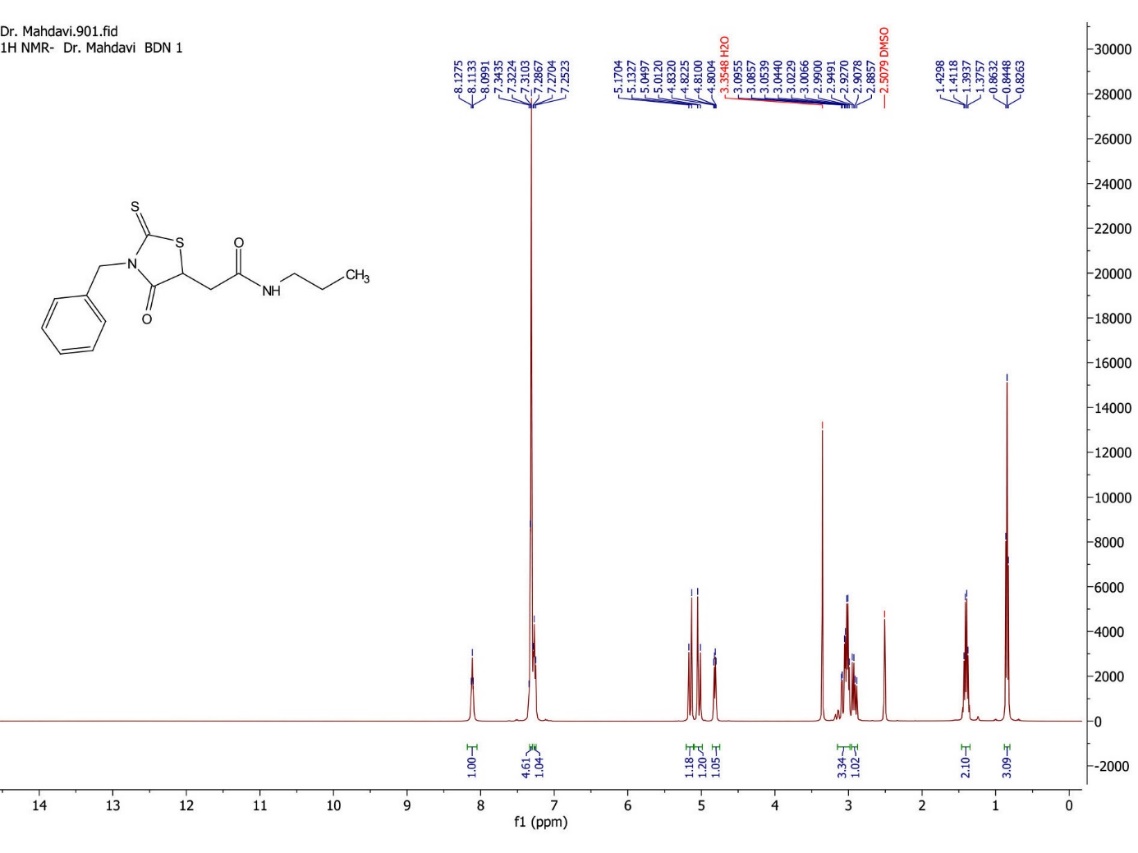


Figure S7. HNMR Spectrum of 2-(3-benzyl-4-oxo-2-thioxothiazolidin-5-yl)-N-propylacetamide (6d)


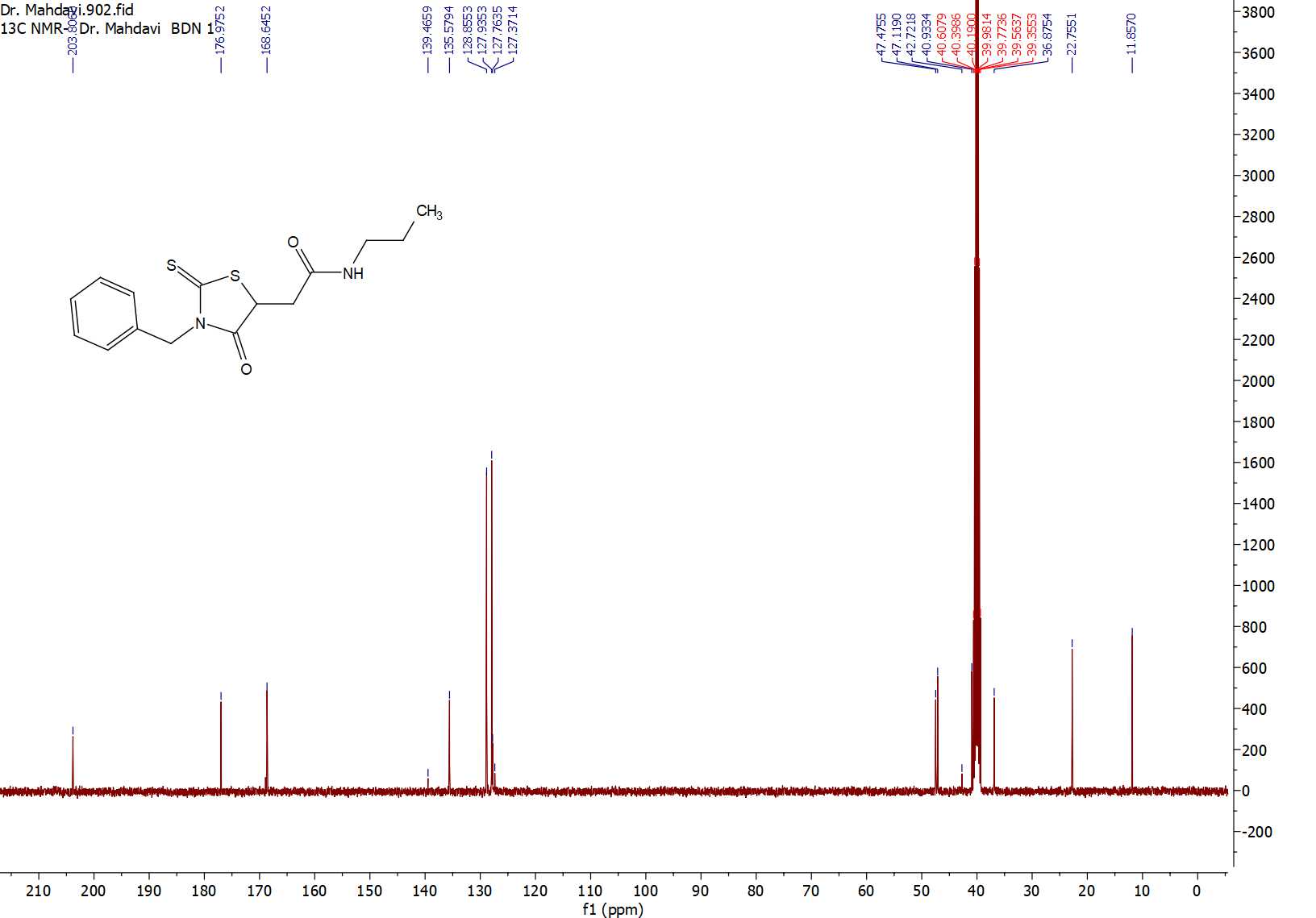


Figure S8. CNMR Spectrum of 2-(3-benzyl-4-oxo-2-thioxothiazolidin-5-yl)-N-propylacetamide (6d)


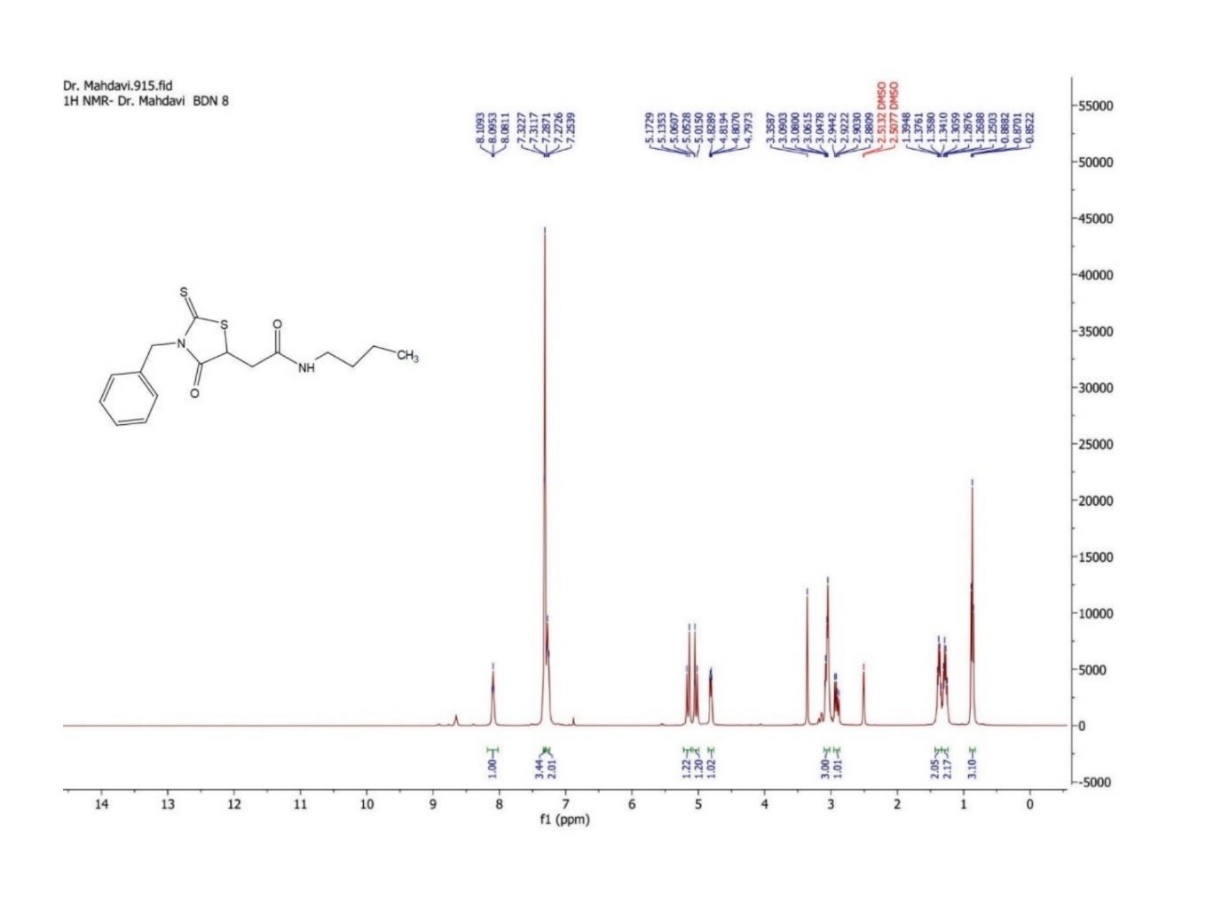


Figure S9. HNMR Spectrum of 2-(3-benzyl-4-oxo-2-thioxothiazolidin-5-yl)-N-butylacetamide (6e)


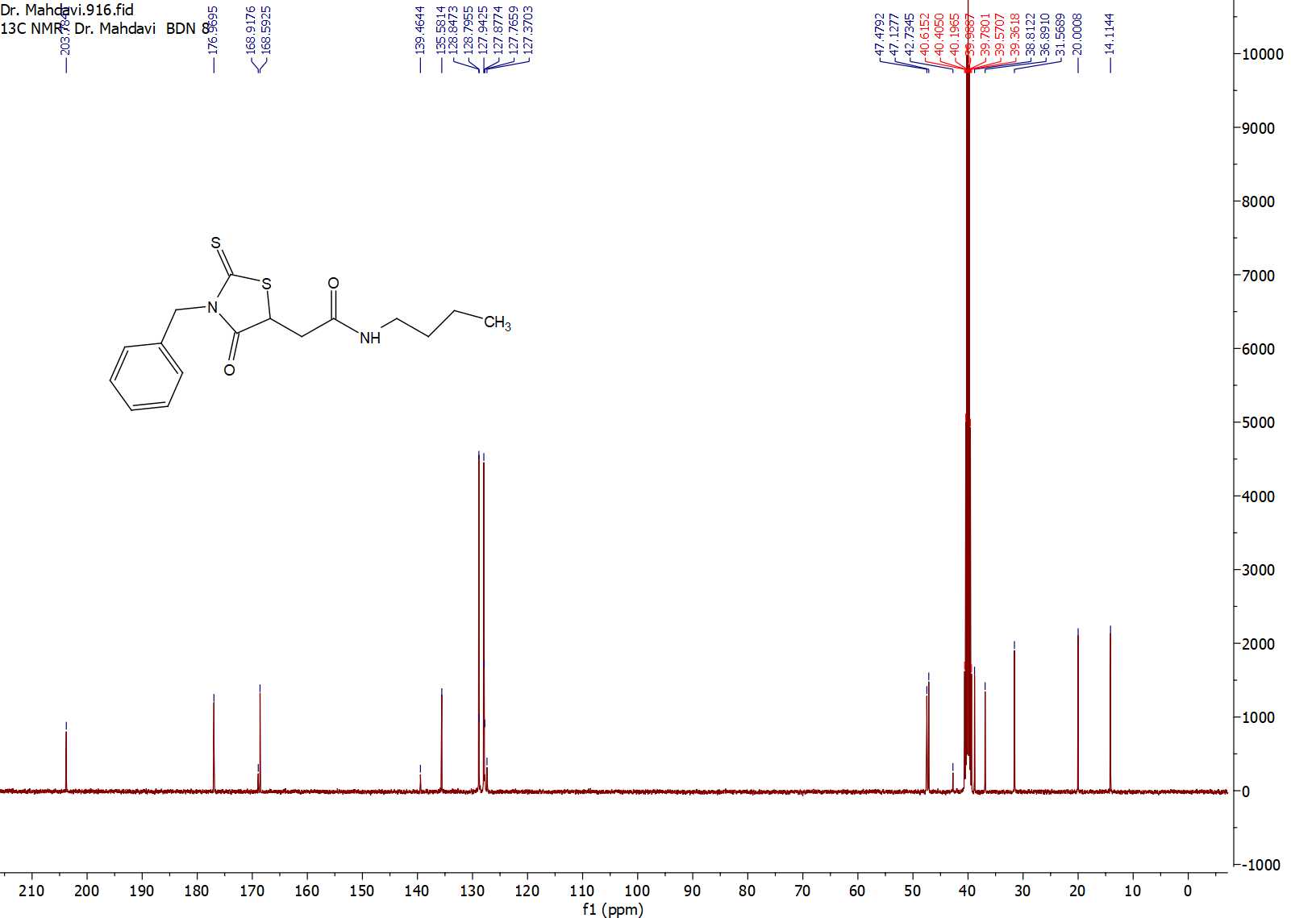


Figure S10. CNMR Spectrum of 2-(3-benzyl-4-oxo-2-thioxothiazolidin-5-yl)-N-butylacetamide (6e)


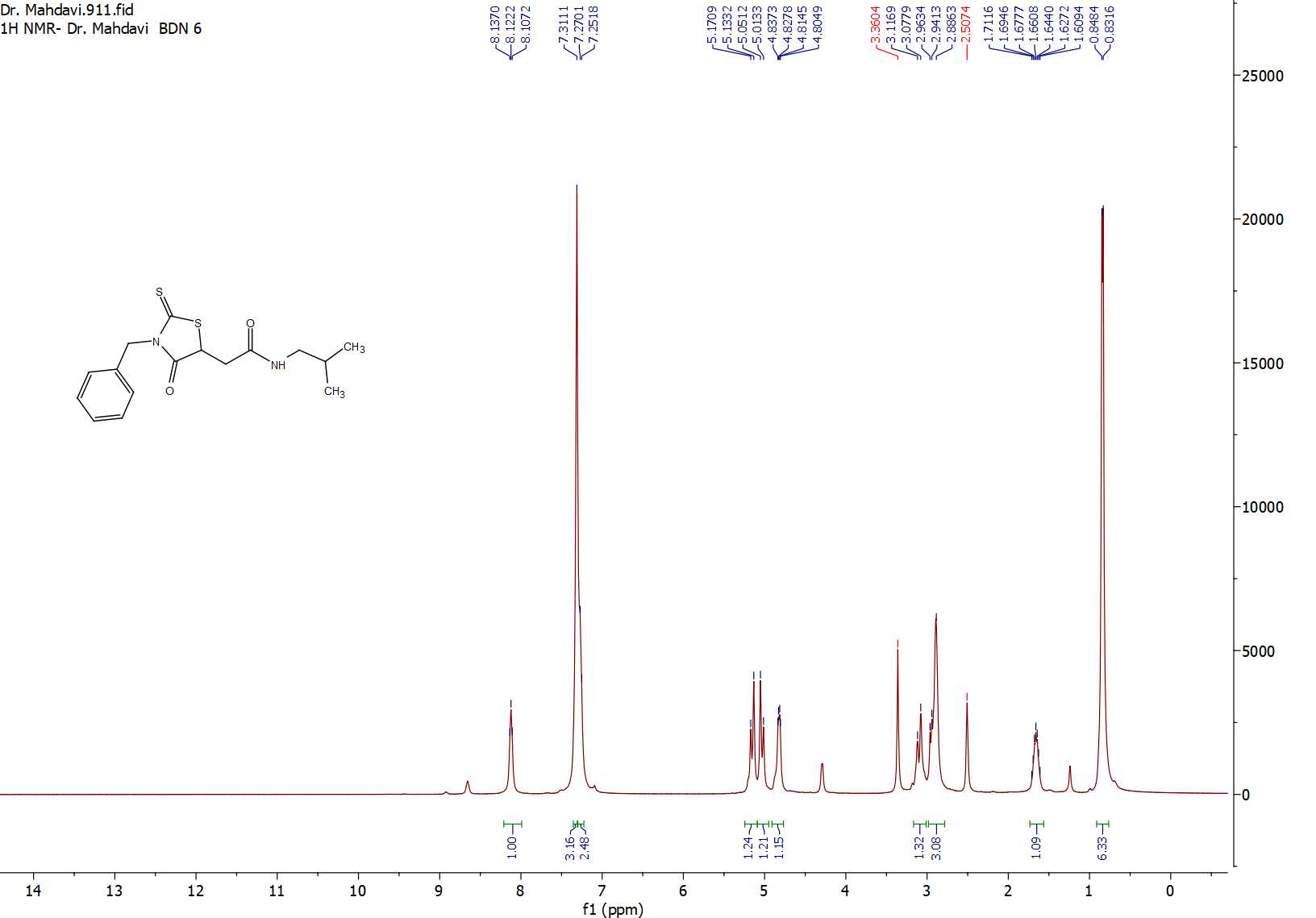


Figure S11. HNMR Spectrum of 2-(3-benzyl-4-oxo-2-thioxothiazolidin-5-yl)-N-isobutylacetamide (6f)


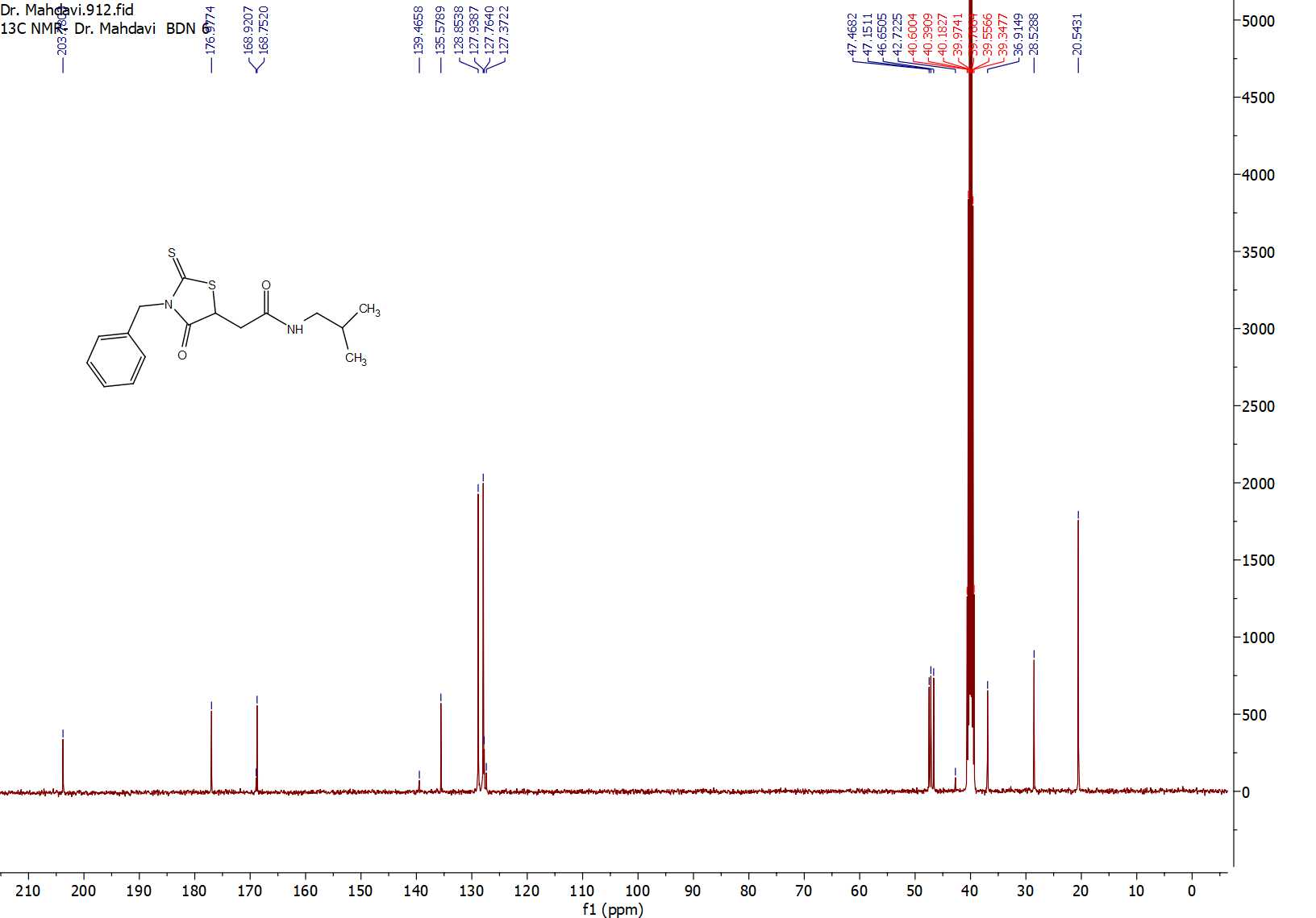


C Figure S12. NMR Spectrum of 2-(3-benzyl-4-oxo-2-thioxothiazolidin-5-yl)-N-isobutylacetamide (6f)


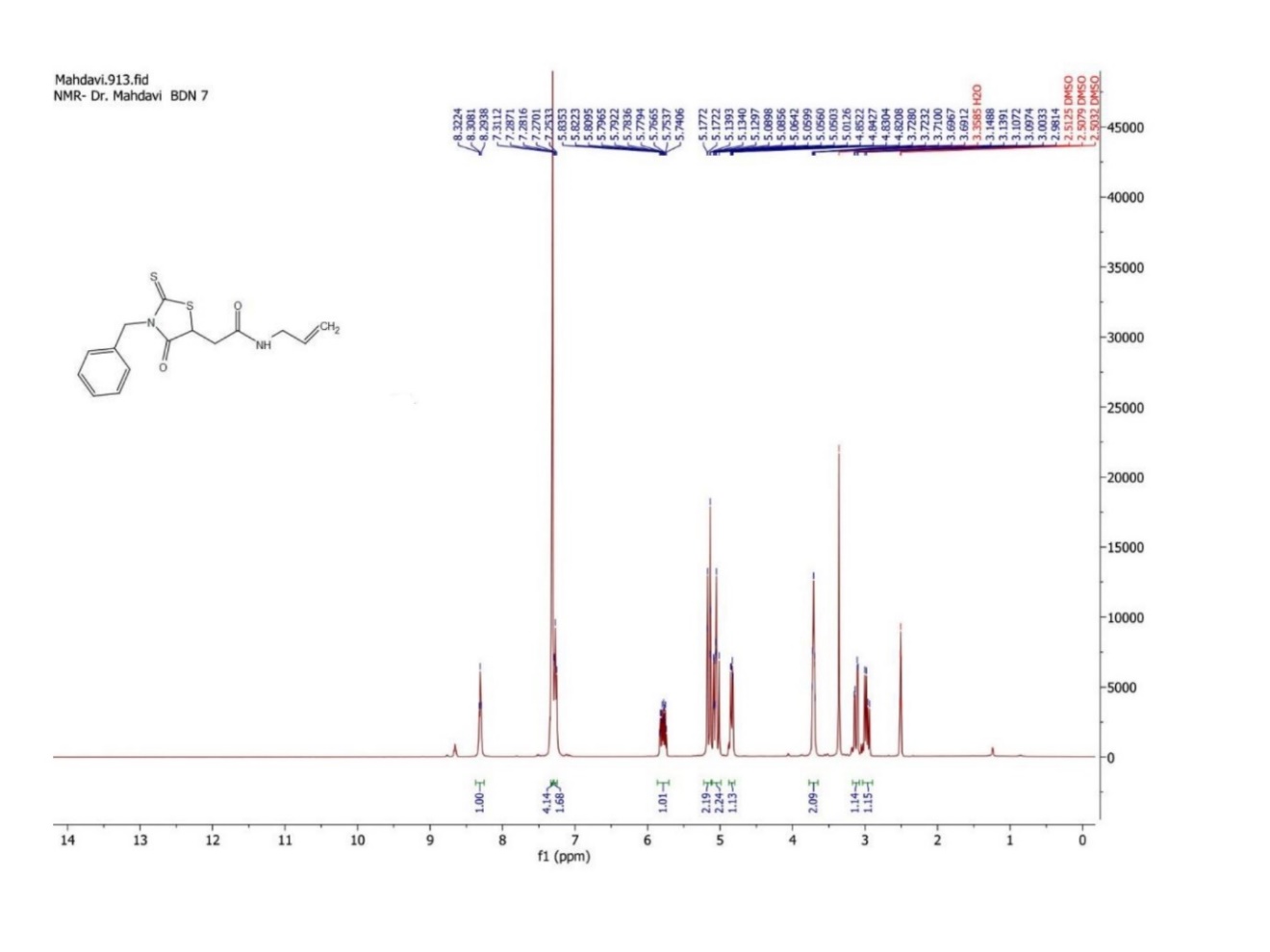


Figure S13. HNMR Spectrum of N-allyl-2-(3-benzyl-4-oxo-2-thioxothiazolidin-5-yl)acetamide (6g)


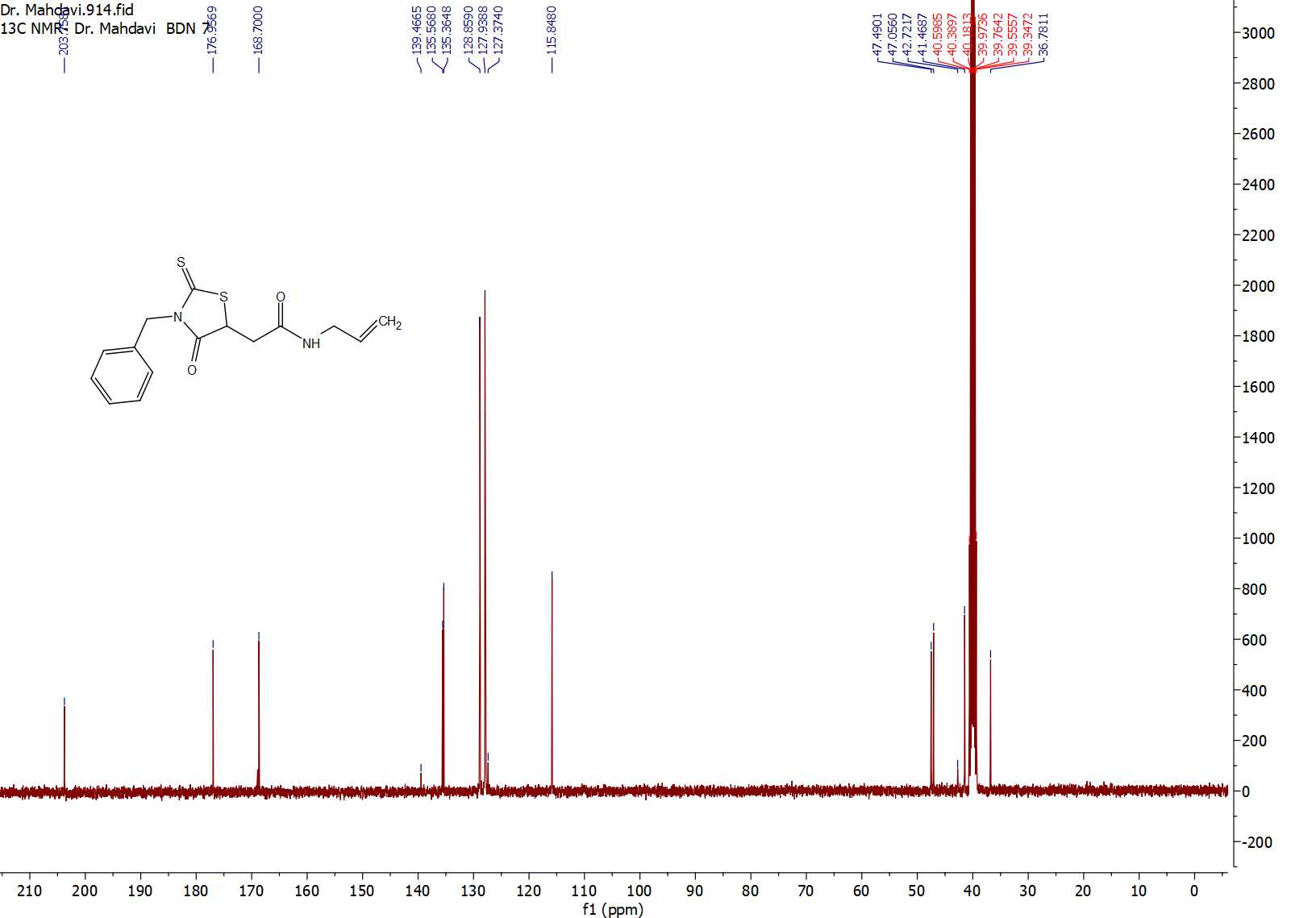


Figure S14. CNMR Spectrum of N-allyl-2-(3-benzyl-4-oxo-2-thioxothiazolidin-5-yl)acetamide (6g)


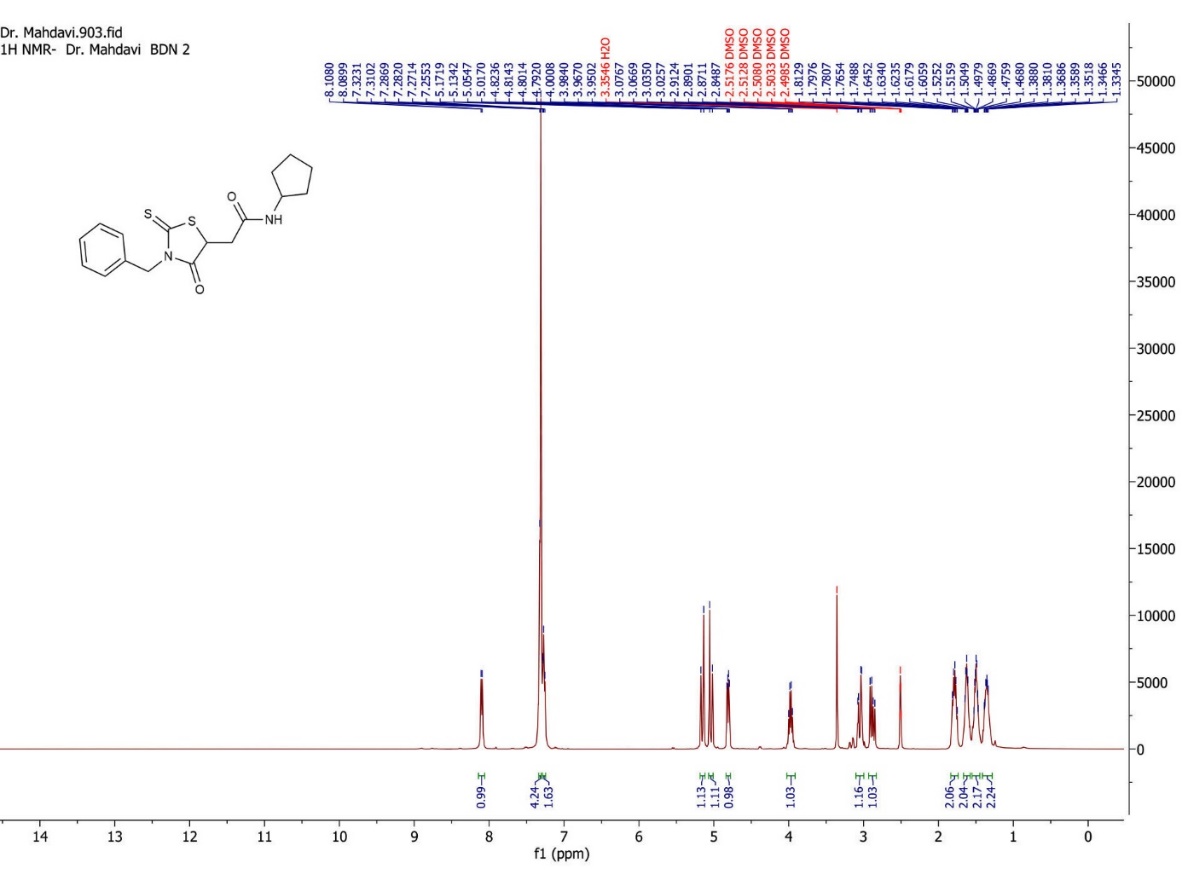


Figure S15. HNMR Spectrum of 2-(3-benzyl-4-oxo-2-thioxothiazolidin-5-yl)-N-cyclopentylacetamide (6h)


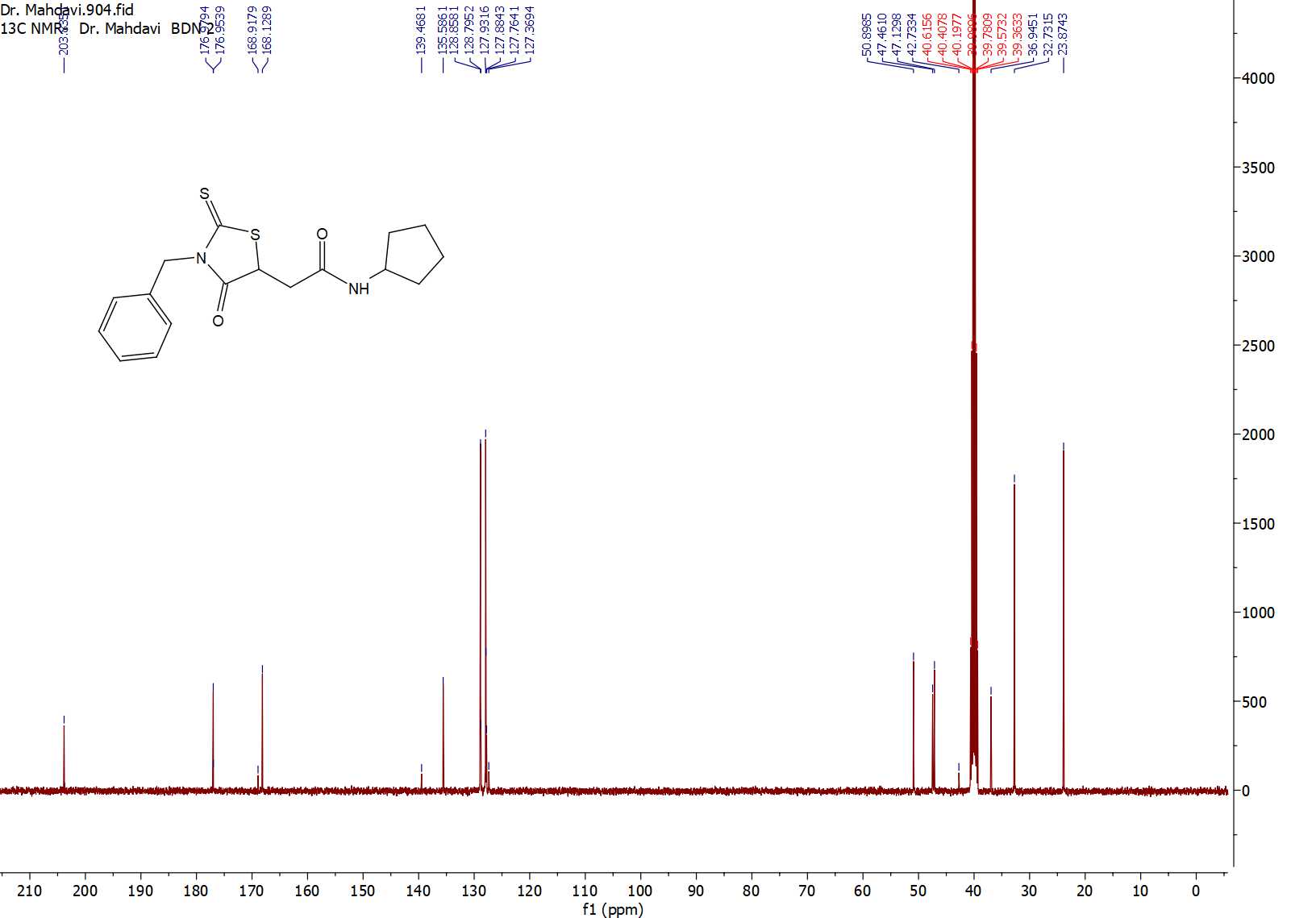


Figure S16. CNMR Spectrum of 2-(3-benzyl-4-oxo-2-thioxothiazolidin-5-yl)-N-cyclopentylacetamide (6h)


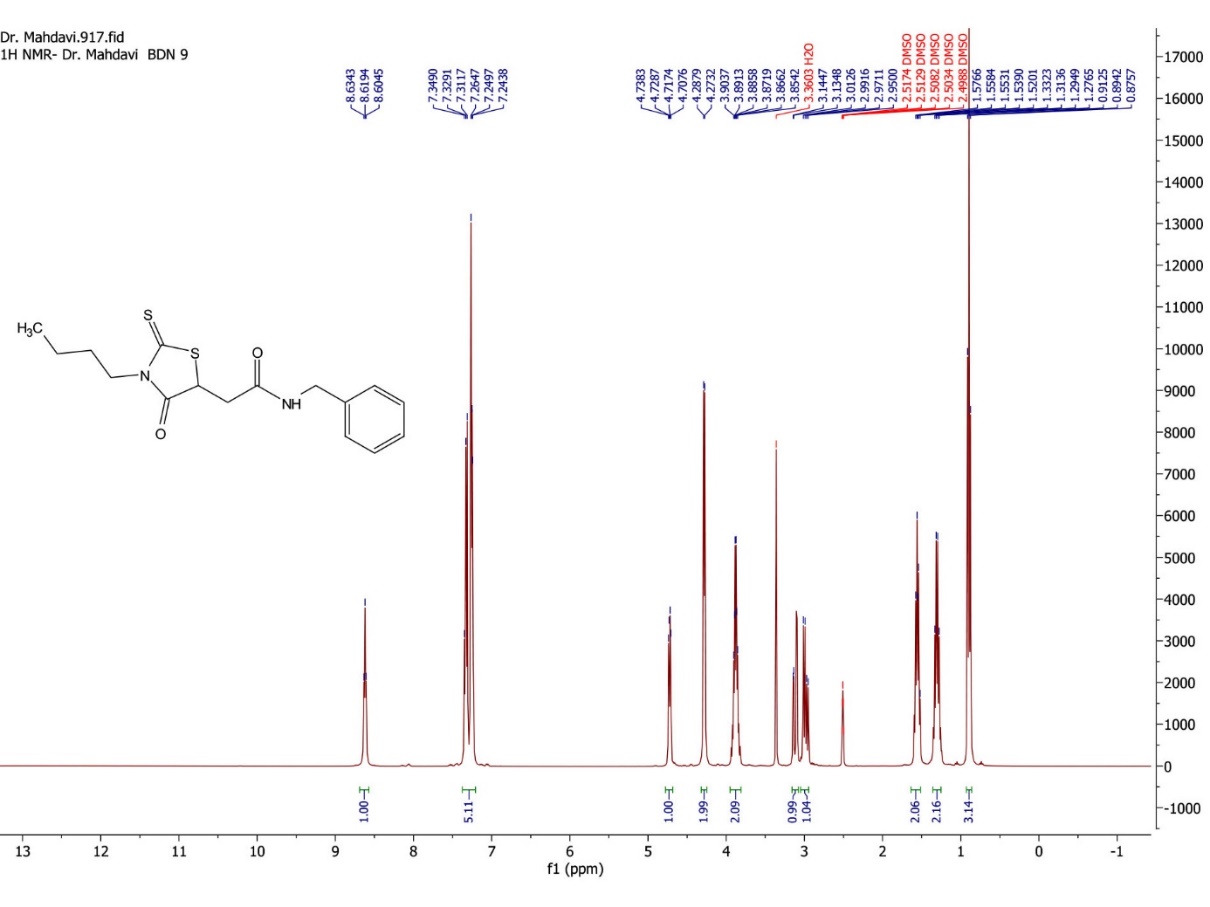


Figure S17. HNMR Spectrum of N-benzyl-2-(3-butyl-4-oxo-2-thioxothiazolidin-5-yl)acetamide (6i)


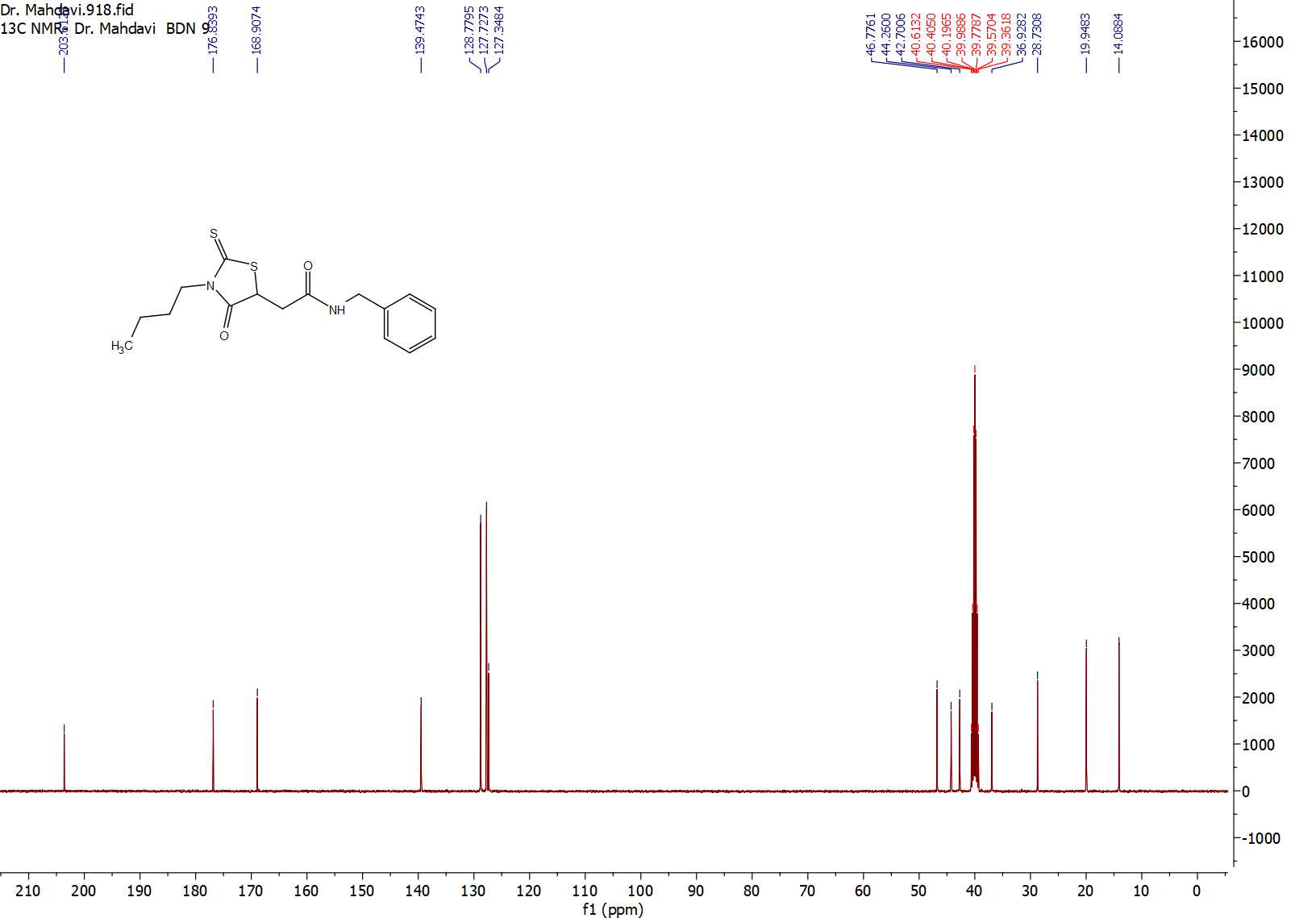


Figure S18. CNMR Spectrum of N-benzyl-2-(3-butyl-4-oxo-2-thioxothiazolidin-5-yl)acetamide (6i)


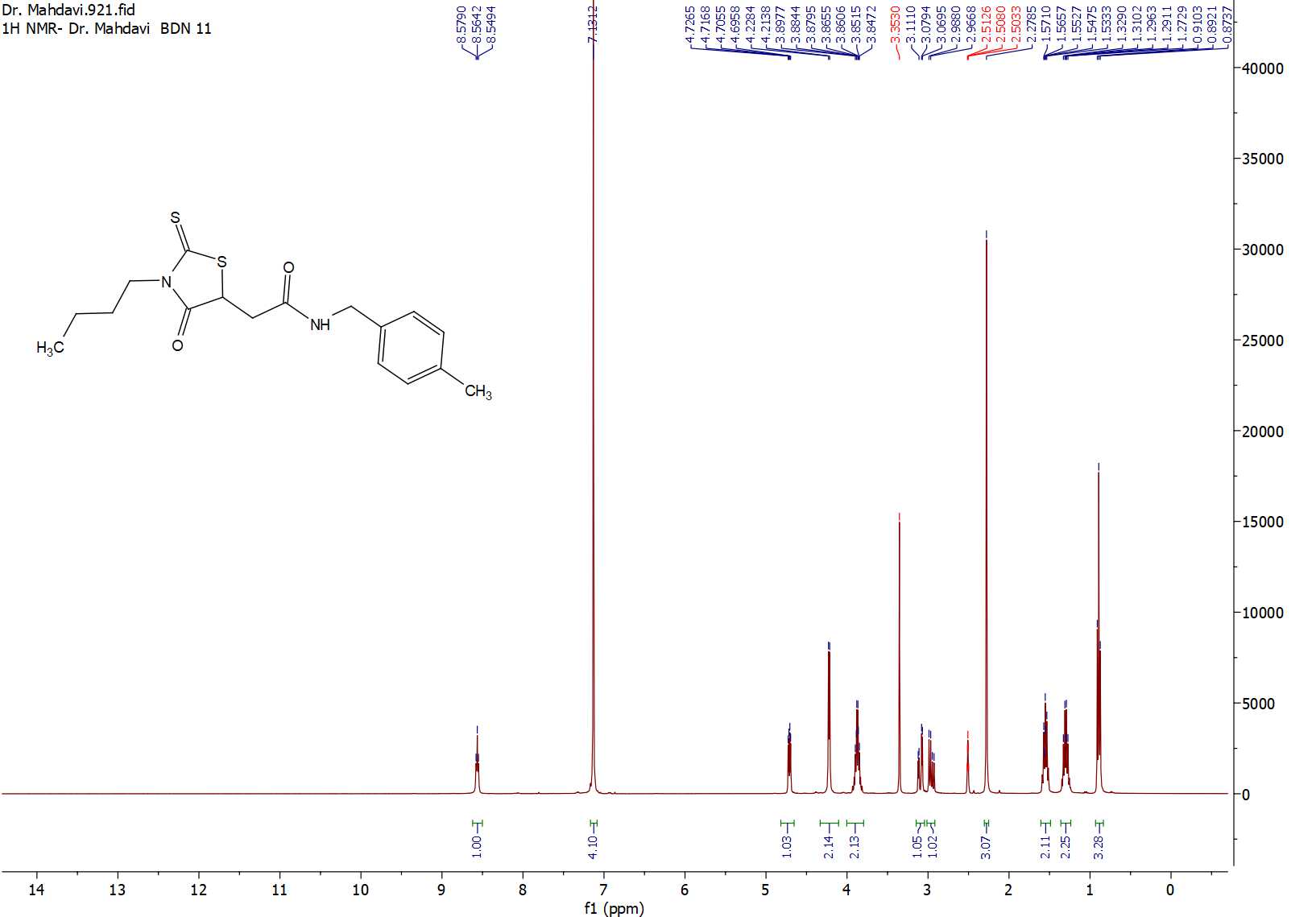


Figure S19. HNMR Spectrum of 2-(3-butyl-4-oxo-2-thioxothiazolidin-5-yl)-N-(4-methylbenzyl)acetamide (6j)


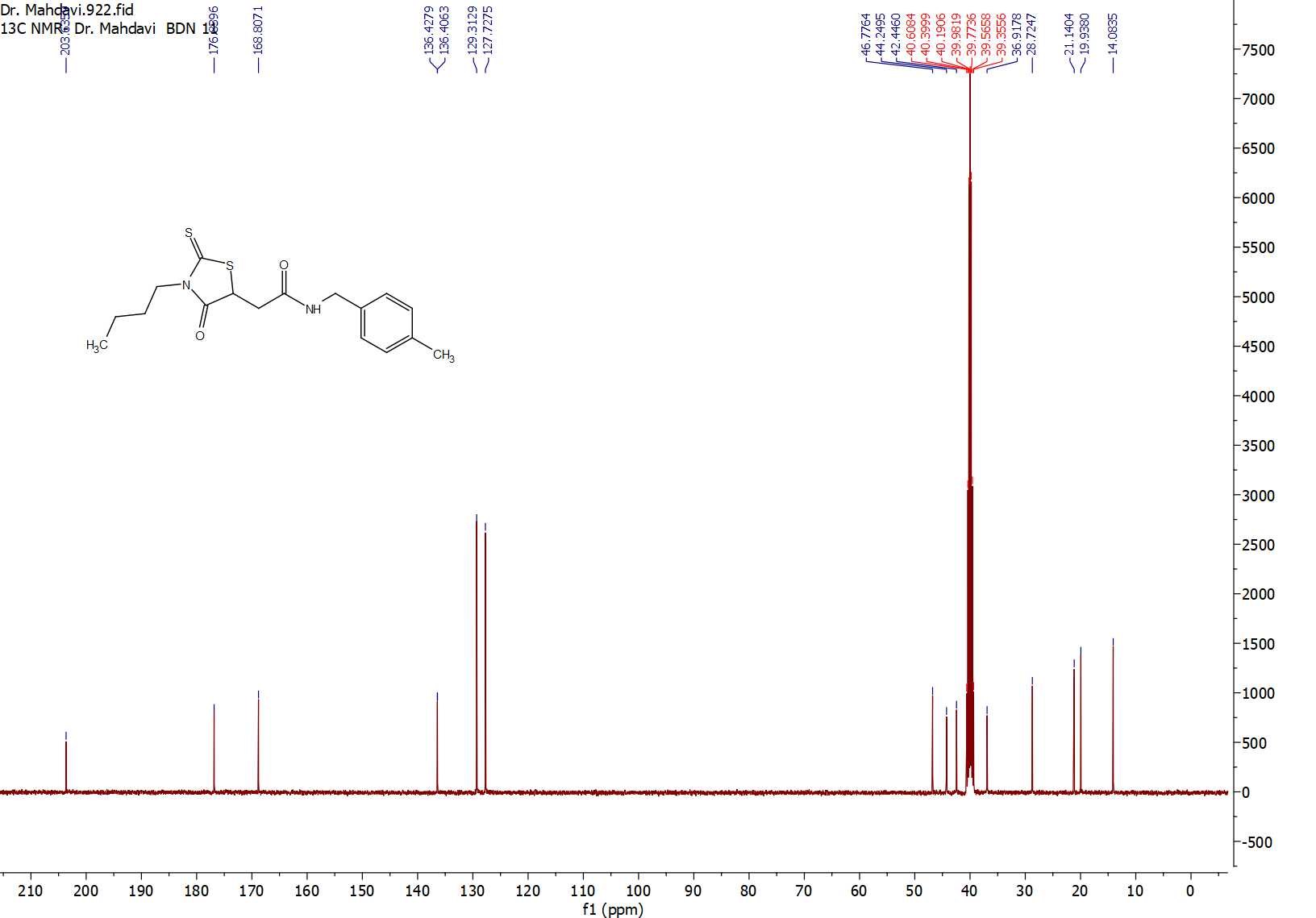


Figure S20. CNMR Spectrum of 2-(3-butyl-4-oxo-2-thioxothiazolidin-5-yl)-N-(4-methylbenzyl)acetamide (6j)


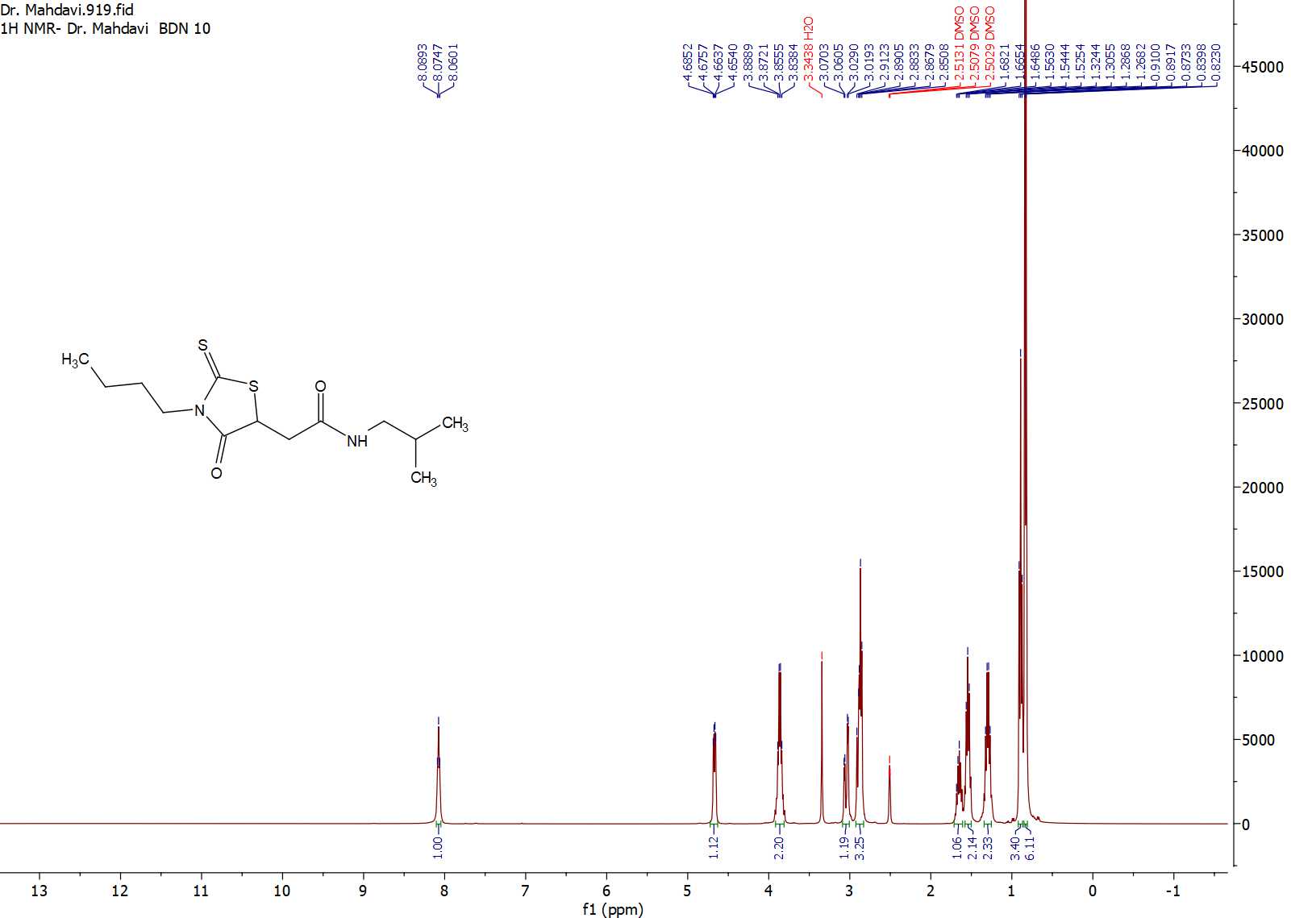


Figure S21. HNMR Spectrum of 2-(3-butyl-4-oxo-2-thioxothiazolidin-5-yl)-N-isobutylacetamide (6k)


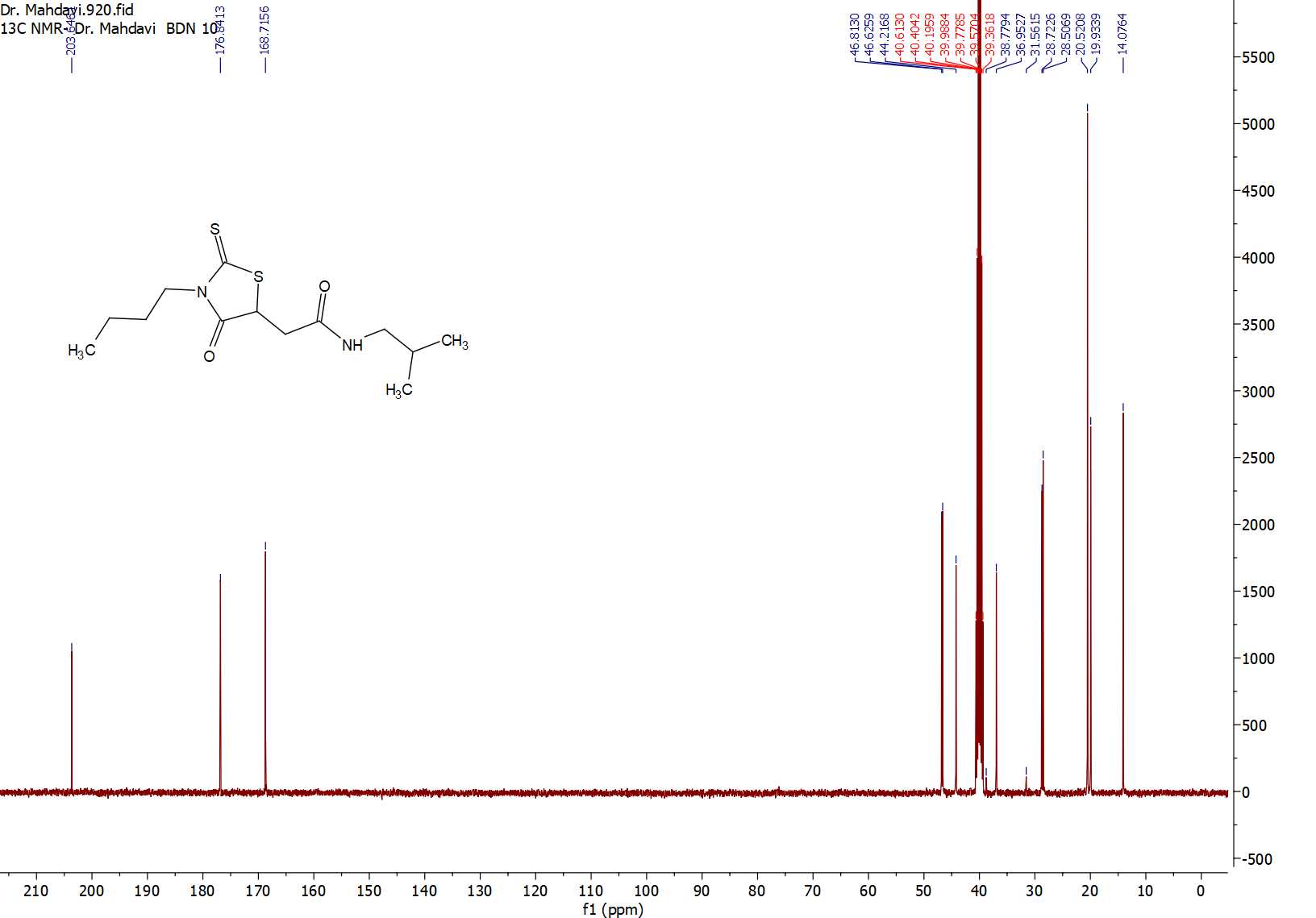


Figure S22. CNMR Spectrum of 2-(3-butyl-4-oxo-2-thioxothiazolidin-5-yl)-N-isobutylacetamide (6k)


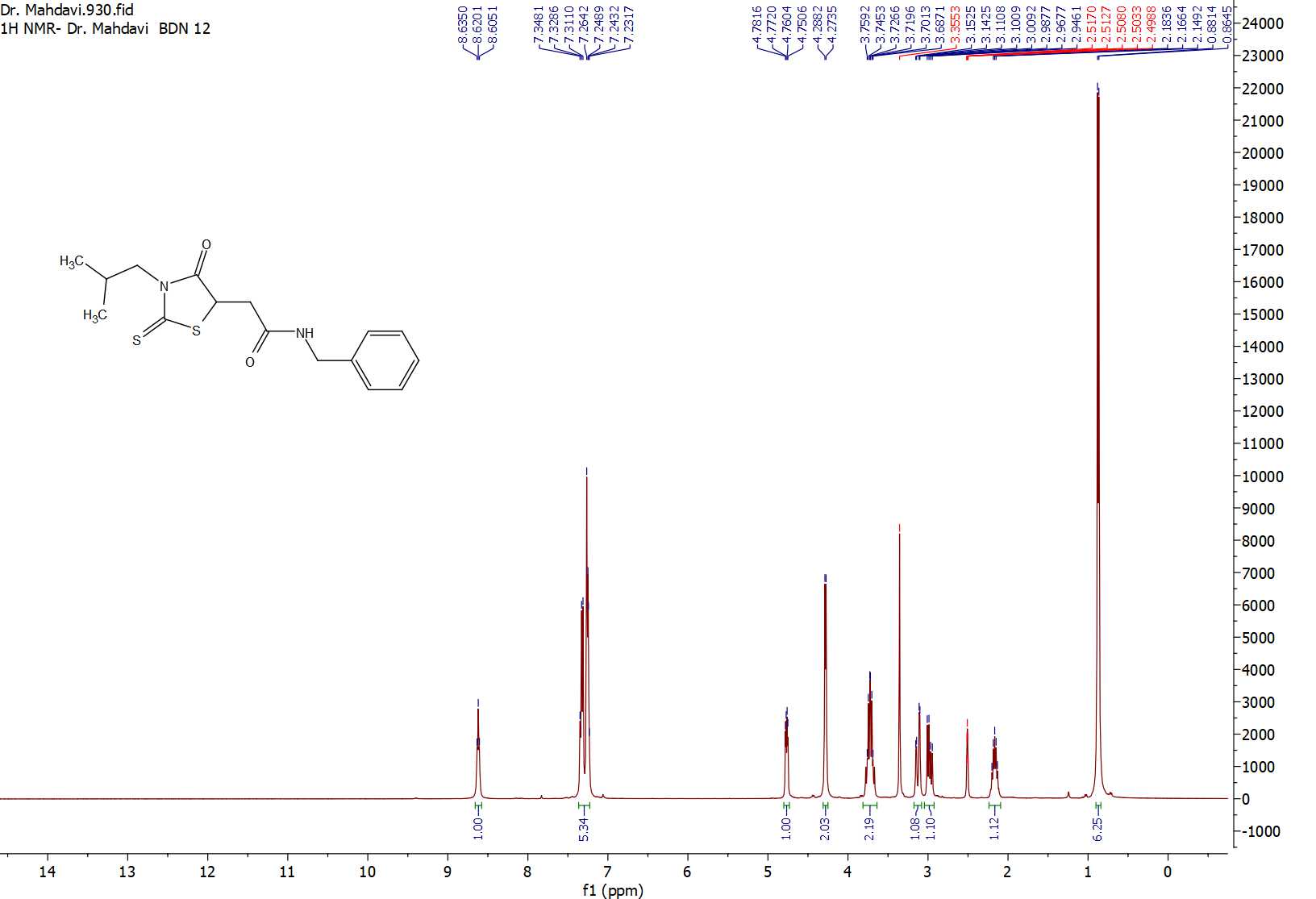


Figure S23. HNMR Spectrum of N-benzyl-2-(3-isobutyl-4-oxo-2-thioxothiazolidin-5-yl)acetamide (6l)


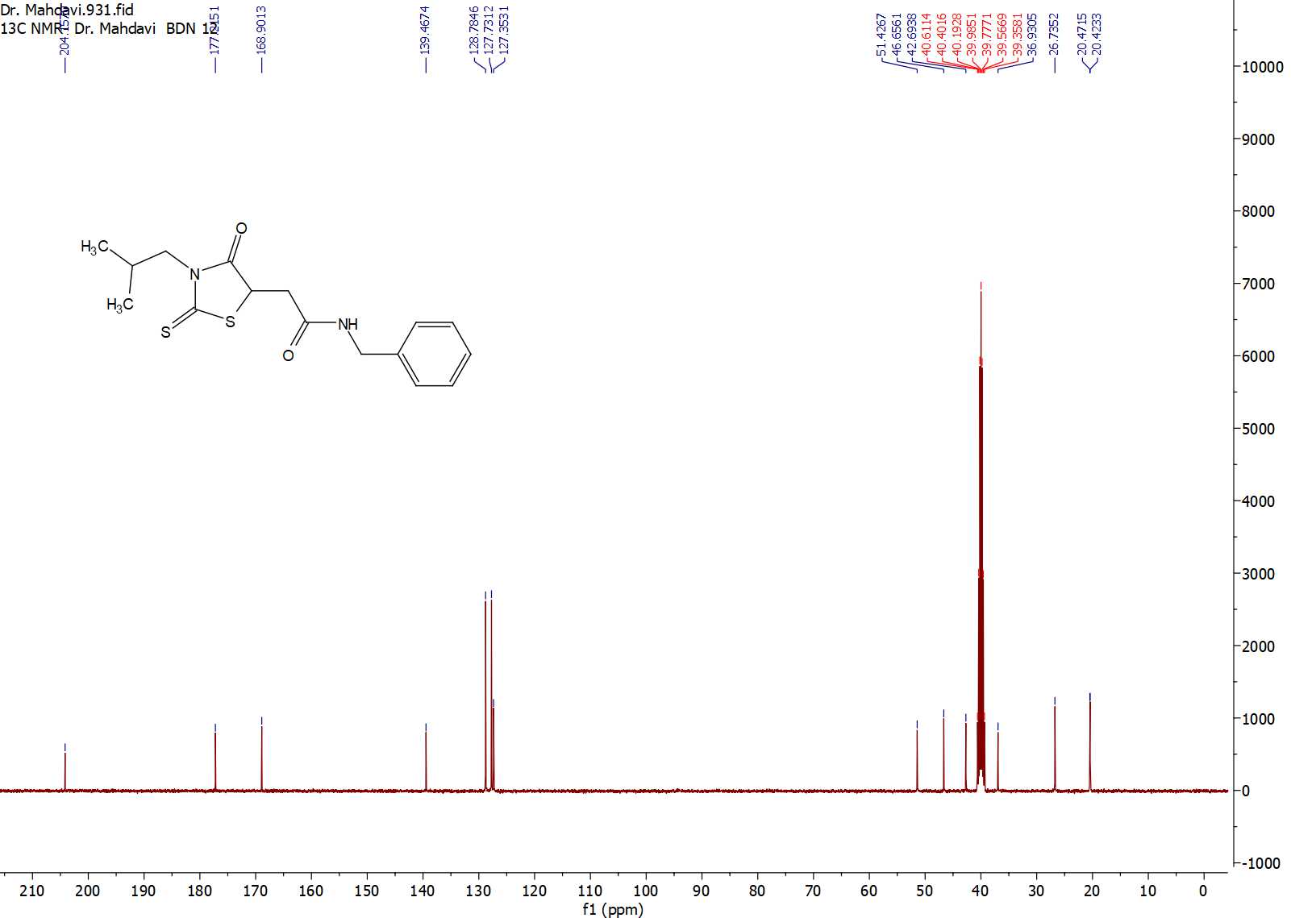


Figure S24. CNMR Spectrum of N-benzyl-2-(3-isobutyl-4-oxo-2-thioxothiazolidin-5-yl)acetamide (6l)


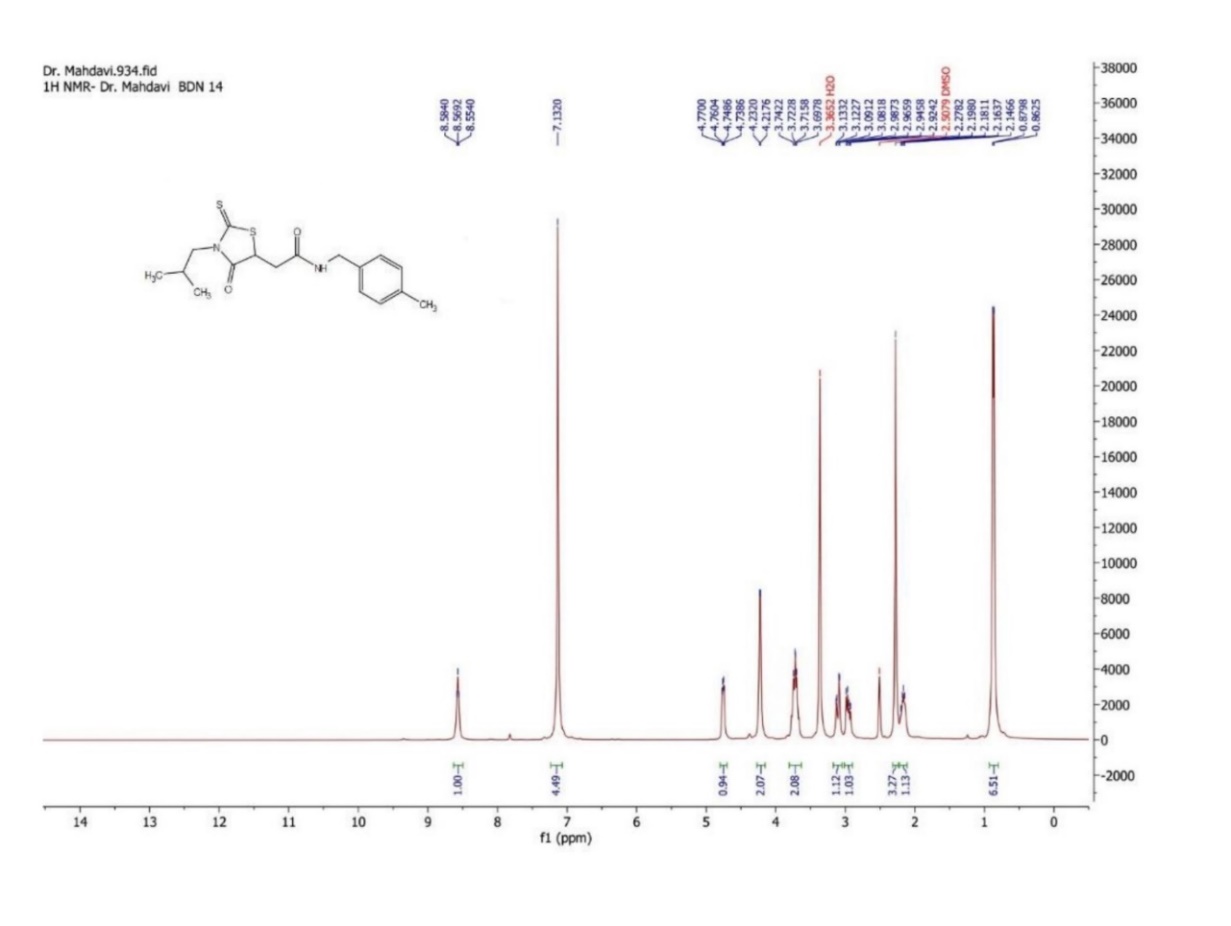


Figure S25. HNMR Spectrum of 2-(3-isobutyl-4-oxo-2-thioxothiazolidin-5-yl)-N-(4-methylbenzyl)acetamide (6m)


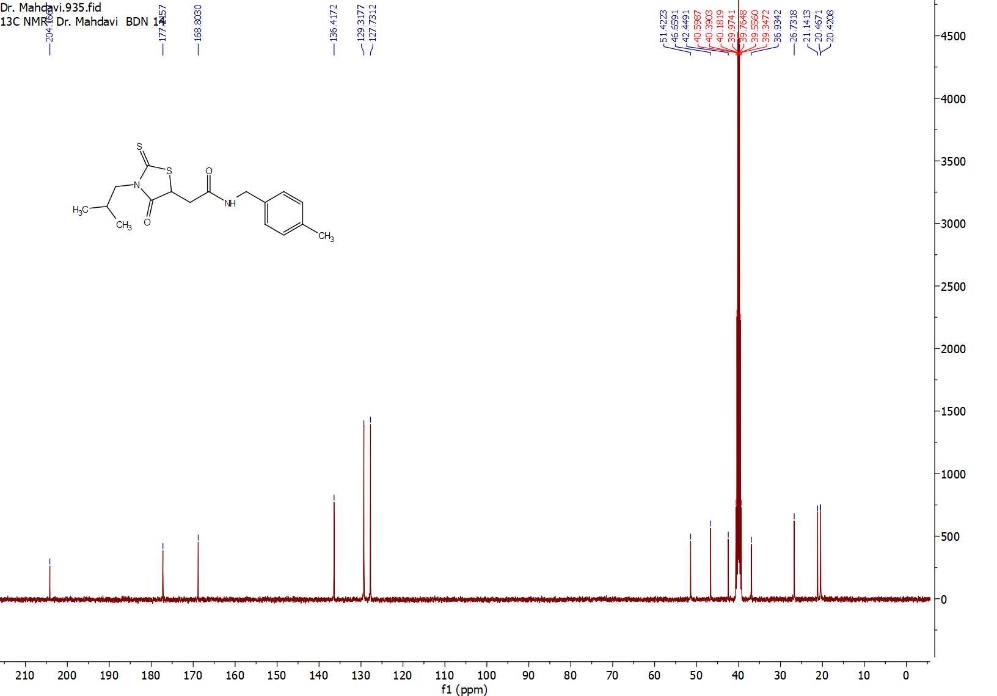


Figure S26. CNMR Spectrum of 2-(3-isobutyl-4-oxo-2-thioxothiazolidin-5-yl)-N-(4-methylbenzyl)acetamide (6m)


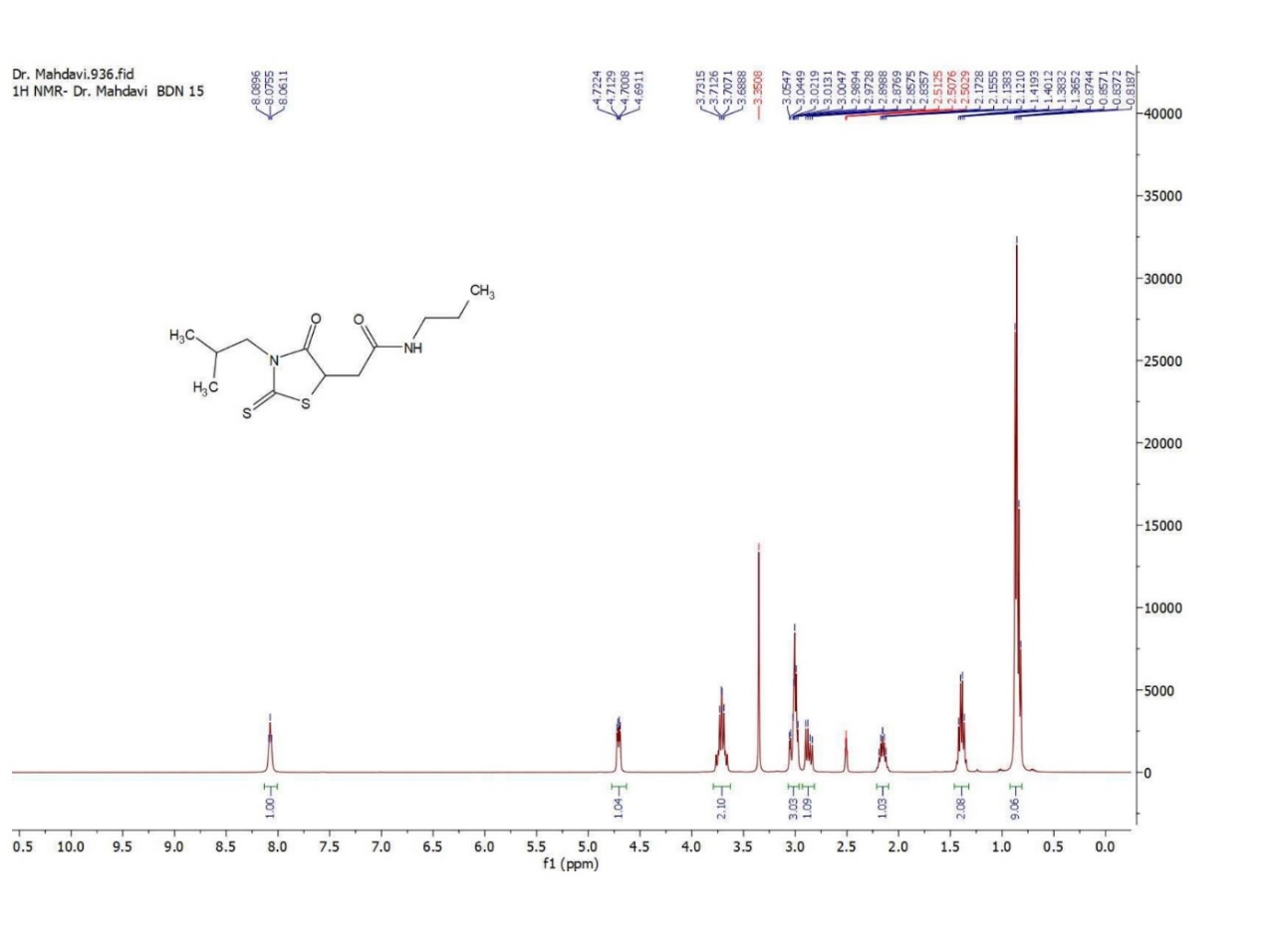


Figure S27. HNMR Spectrum of 2-(3-isobutyl-4-oxo-2-thioxothiazolidin-5-yl)-N-propylacetamide (6n)


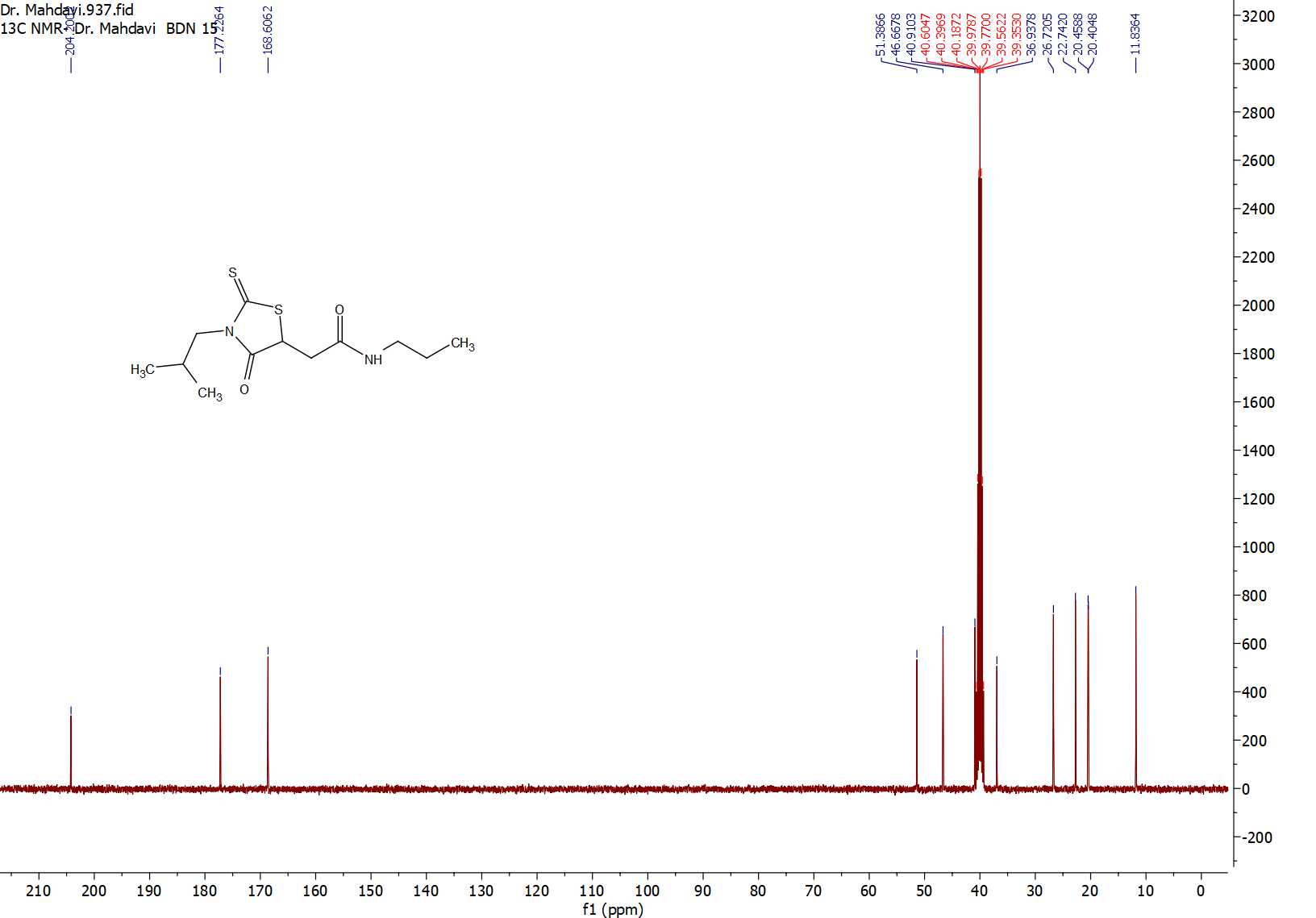


Figure S28. CNMR Spectrum of 2-(3-isobutyl-4-oxo-2-thioxothiazolidin-5-yl)-N-propylacetamide (6n)


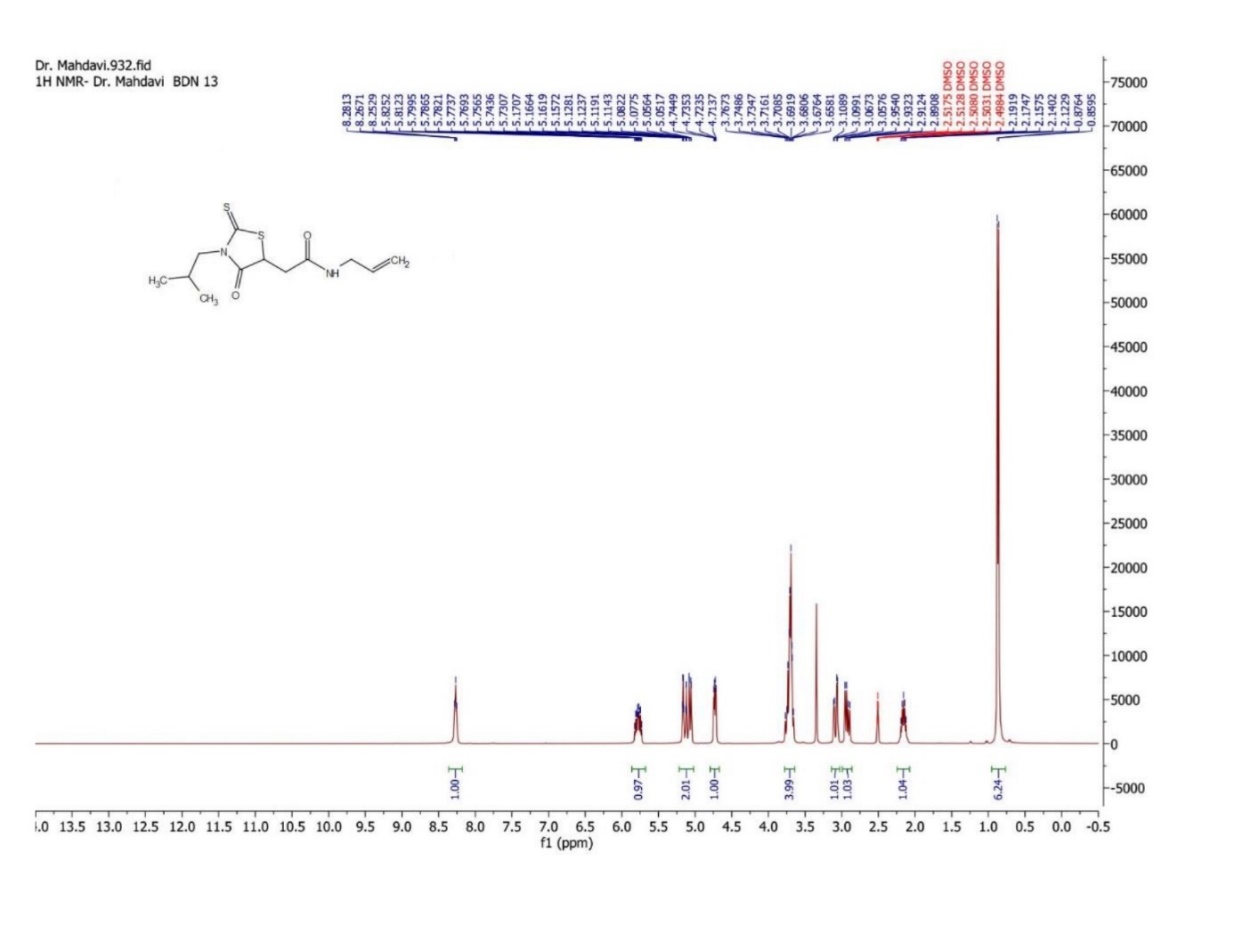


Figure S29. HNMR Spectrum of N-allyl-2-(3-isobutyl-4-oxo-2-thioxothiazolidin-5-yl)acetamide (6o)


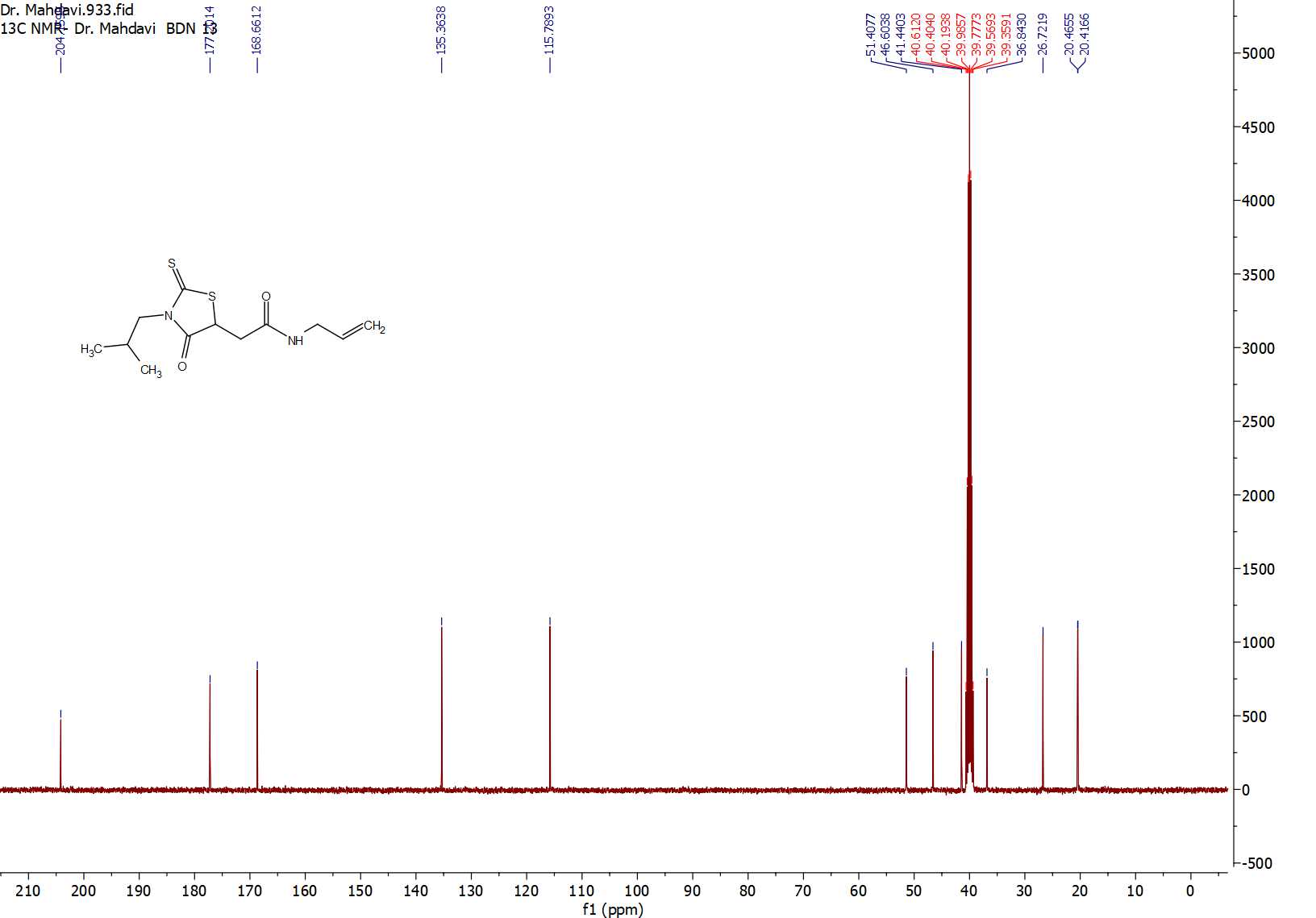
 CNMR Figure S30. Spectrum of N-allyl-2-(3-isobutyl-4-oxo-2-thioxothiazolidin-5-yl)acetamide (6o)
